# Immunogenicity and real-world effectiveness of COVID-19 vaccines in Lebanon: Insights from primary and booster schemes, variants, infections, and hospitalization

**DOI:** 10.1371/journal.pone.0306457

**Published:** 2024-09-13

**Authors:** Rima Moghnieh, Wajdi Haddad, Nayla Jbeily, Salam El-Hassan, Shadi Eid, Hicham Baba, Marilyne Sily, Yara Saber, Dania Abdallah, Abdul Rahman Bizri, Mohamed H. Sayegh

**Affiliations:** 1 Division of Infectious Diseases, Department of Internal Medicine, Lebanese American University Medical Center-Rizk Hospital, Beirut, Lebanon; 2 Department of Internal Medicine, Central Military Hospital, Military Healthcare, Lebanese Army, Beirut, Lebanon; 3 Head of Laboratory Department, FMPS Holding S.A.L., Beirut, Lebanon; 4 Nursing Office, Makassed General Hospital, Beirut, Lebanon; 5 Gilbert and Rose-Marie Chagoury School of Medicine, Lebanese American University, Byblos, Lebanon; 6 Laboratory Department, FMPS Holding S.A.L., Beirut, Lebanon; 7 Pharmacy Department, Makassed General Hospital, Beirut, Lebanon; 8 American University of Beirut, Beirut, Lebanon; 9 Department of Health and Human Services, GAP Solutions (Contract No. 75N93019D00026), National Institute of Allergy and Infectious Diseases, National Institutes of Health, United States of America; King Saud University College of Medicine, SAUDI ARABIA

## Abstract

In this study, we conducted a case-control investigation to assess the immunogenicity and effectiveness of primary and first booster homologous and heterologous COVID-19 vaccination regimens against infection and hospitalization, targeting variants circulating in Lebanon during 2021–2022. The study population comprised active Lebanese military personnel between February 2021 and September 2022. Vaccine effectiveness (VE) against laboratory-confirmed SARS-CoV-2 infection and associated hospitalization was retrospectively determined during different variant-predominant periods using a case-control study design. Vaccines developed by Sinopharm, Pfizer, and AstraZeneca as well as Sputnik V were analyzed. Prospective assessment of humoral immune response, which was measured based on the SARS-CoV-2 antispike receptor binding domain IgG titer, was performed post vaccination at various time points, focusing on Sinopharm and Pfizer vaccines. Statistical analyses were performed using IBM SPSS and GraphPad Prism. COVID-19 VE remained consistently high before the emergence of the Omicron variant, with lower estimates during the Delta wave than those during the Alpha wave for primary vaccination schemes. However, vaccines continued to offer significant protection against infection. VE estimates consistently decreased for the Omicron variant across post-vaccination timeframes and schemes. VE against hospitalization declined over time and was influenced by the variant. No breakthrough infections progressed to critical or fatal COVID-19. Immunogenicity analysis revealed that the homologous Pfizer regimen elicited a stronger humoral response than Sinopharm, while a heterologous Sinopharm/Pfizer regimen yielded comparable results to the Pfizer regimen. Over time, both Sinopharm’s and Pfizer’s primary vaccination schemes exhibited decreased humoral immunity titers, with Pfizer being a more effective booster than Sinopharm. This study, focusing on healthy young adults, provides insights into VE during different pandemic waves. Continuous research and monitoring are essential for understanding vaccine-mediated immune responses under evolving circumstances.

## 1. Introduction

The COVID-19 pandemic, although expected by some scientists, took the world by surprise and had a catalytic impact on the lives of individuals worldwide [1]. It fragmented human memory into eras before, during, and after the pandemic. For the past few years, it has been an unprecedented global health crisis, profoundly impacting the lives of individuals and communities worldwide, exacerbating existing crises and humanitarian needs, and drawing all nations’ frailties and inequities into sharp focus [2]. It has also triggered a socioeconomic crisis of unprecedented proportion [3]. This crisis parallels past events in history, such as the Spanish flu pandemic, which claimed millions of lives, and the subsequent economic crisis that paved the way for World War II [4, 5].

The COVID-19 pandemic has brought to the forefront the risk of major disease outbreaks and underscored countries’ lack of preparedness to fight them, even the highly resourced ones [6–8]. Moreover, the pandemic highlighted previously existing weaknesses, including disparities in healthcare access and ineffective communication between public health and healthcare delivery systems [6–8].

Pandemic preparedness and disease surveillance anchored in strong healthcare delivery systems that reach all people, especially the most vulnerable, are crucial to ensure better protection from major disease outbreaks [6–8].

Vaccine science and the pharmaceutical industry are at the forefront of pandemic preparedness. Nevertheless, the use of vaccines against COVID-19 has shown that gaps regarding our comprehension of vaccines and their interaction with the dynamic nature of microorganisms, namely viruses, still exist [9, 10]. During the pandemic, different parts of the world adopted various vaccine platforms, leading to a significant amount of confusion and speculation about which vaccine or vaccine platform is superior. To shed light on this matter, laboratory-based immunogenicity studies were conducted, followed by randomized controlled trials to assess the efficacy of these vaccines [9, 11, 12]. The results of these studies appeared promising, reporting high levels of efficacy [9–11]. However, when it came to real-world effectiveness, conflicting information emerged in comparison with the results of immunogenicity and efficacy trials [13]. This discrepancy may be attributed to several factors, such as these trials were conducted at different times and countries during the pandemic and with different newly emerging and circulating SARS-CoV-2 variants, which may not align well with the vaccines available at a particular time and place [13].

In Lebanon, the national COVID-19 vaccination campaign started in mid-February, 2021. Several vaccines using various platforms received emergency use authorization from the Lebanese Ministry of Health (MoH). These vaccines included those from Pfizer-BioNTech (BNT162b2), Sinopharm (BBIBP-CorV), Gamaleya Sputnik V (Gam-COVID-Vac), and AstraZeneca (ChAdOx1 nCoV-19). These vaccines were distributed through the Lebanese MoH, private sector procurement, and donations. This unique situation provided an opportunity to assess the immunogenicity and effectiveness of these vaccines, both as primary vaccinations and first booster regimens, against COVID-19 variants prevalent in Lebanon during 2021 and 2022.

In this study, we conducted a case-control investigation to determine the effectiveness of available primary and initial booster COVID-19 vaccination regimens against infection using notable variants of concern that predominantly circulated in Lebanon during different periods. These variants included the Alpha (B.1.1.7), Delta (B.1.617.2), and Omicron (B.1.1.529) variants along with its sublineages BA.1, BA.1.1, BA.2, BA.4, and BA.5. We also assessed vaccine effectiveness (VE) in preventing hospitalization during these timeframes. In addition, we measured the SARS-CoV-2 antispike receptor binding domain (RBD) immunoglobulin G (anti-S-IgG) titer as a marker of the immunogenicity of certain vaccine regimens to compare the potential level of protection against infection among vaccinees.

## 2. Materials and methods

### 2.1 Study population, data sources, and study design

This study is divided into two parts:

- A retrospective analysis dealing with the effectiveness of different homologous and heterologous vaccination schemes at various time points. Vaccines included in the analysis were as follows:
  - Sinopharm (BBIBP-CorV)
  - Pfizer (BNT162b2)
  - Sputnik V (Gam-COVID-Vac)
  - AstraZeneca (ChAdOx1 nCoV-19)
- A prospective analysis dealing with the immunogenicity of different homologous and heterologous vaccination schemes at different time points.

### 2.2 VE against COVID-19 infection and hospitalization

This retrospective cohort case-control study examined the effectiveness of two-dose primary vaccination schemes (2× Sinopharm, 2× Pfizer, 2× Sputnik V, 2× AstraZeneca, or 1× Sinopharm/1× Pfizer) and homologous (2× Sinopharm/1× Sinopharm or 2× Pfizer/1× Pfizer) or heterologous (2× Sinopharm/1× Pfizer, 2× Sputnik V/1× Pfizer, or 2× Pfizer/1× Sinopharm) first booster vaccination schemes against laboratory-confirmed SARS-CoV-2 infections and subsequent hospitalization during the Alpha (B.1.1.7), Delta (B.1.617.2), and Omicron (B.1.1.529, with its sublineages BA.1, BA.2, BA.4, and BA.5) variants that circulated in Lebanon between 2021 and 2022, compared with unvaccinated individuals without previous exposure to COVID-19.

The study population comprised active Lebanese military personnel between February 2021 and September 2022, who were followed longitudinally throughout the study period. Data were retrieved using the Lebanese Ministry of Defense (MoD) COVID-19 electronic healthcare database. The study included personnel who had received the mentioned vaccination schemes as well as unvaccinated personnel, both with and without previous exposure to COVID-19. Personnel who were partially vaccinated or who received vaccination schemes other than those previously mentioned were excluded from the analysis. Data, including SARS-CoV-2 polymerase chain reaction (PCR) testing results; COVID-19 vaccination history; clinical infection and hospitalization; and other demographic and comorbidity information, including cardiovascular disease, pulmonary disease, kidney disease, diabetes mellitus, malignancy, and obesity, were extracted by the principal investigators from the digital health information database.

Since the onset of the pandemic, the MoD followed the Lebanese MoH recommendations and guidelines regarding SARS-CoV-2 PCR testing, contact tracing, isolation and quarantine, absenteeism, and management and treatment of infection, which were in line with the recommendations of the United States (US) Centers for Disease Control and Prevention (CDC) and the World Health Organization (WHO) [14–17]. In alignment with the national vaccination campaign, the MoD initiated COVID-19 vaccinations for military personnel in mid-February 2021 and began administering booster doses in November 2021. The MoD followed the Lebanese MoH’s guidance on COVID-19 vaccination schedules, which were based on the recommendations of the CDC and WHO [15, 17]. Personnel received vaccines at military treatment facilities and non-military vaccination centers. Regarding the type of vaccine administered, there was no preference, as all were donated to the Lebanese military institution.

A PCR-positive swab, irrespective of the reason for PCR testing or the presence of symptoms, was used to define laboratory-confirmed SARS-CoV-2 infections. Breakthrough SARS-CoV-2 infections were considered to occur at least 14 days after the second dose of the primary vaccination regimen listed above and at least 7 days after receiving the booster dose [18–20]. A positive SARS-CoV-2 PCR test within 3 weeks of a previous positive SARS-CoV-2 PCR test was excluded and not counted as a new infection. Infection severity classification followed the WHO guidelines for COVID-19 case severity (acute-care hospitalizations) [14].

In Lebanon, the COVID-19 pandemic was characterized by the dominance of three SARS-CoV-2 variants during 2021 and 2022, as determined by analyzing the Lebanese dataset in GISAID [21–24]:

- The Alpha (B.1.1.7) variant predominantly circulated until the end of May 2021.
- By the end of July 2021, the Delta (B.1.617.2) variant had completely replaced the Alpha (B.1.1.7) variant.
- The Delta (B.1.617.2) variant, in turn, was gradually replaced by the Omicron (B.1.1.529, sublineages BA.1 and BA.1.1) variant in the second week of November 2021 until the end of December 2021.
- The Omicron (B.1.1.529, sublineages BA.1, BA.1.1, or BA.2) variant predominantly circulated between January 2022 and May 2022.
- By mid-June 2022, the Omicron variant of concern (B.1.1.529, sublineages BA.4 or BA.5) had completely replaced the previously mentioned sublineages.

**Fig 1:**
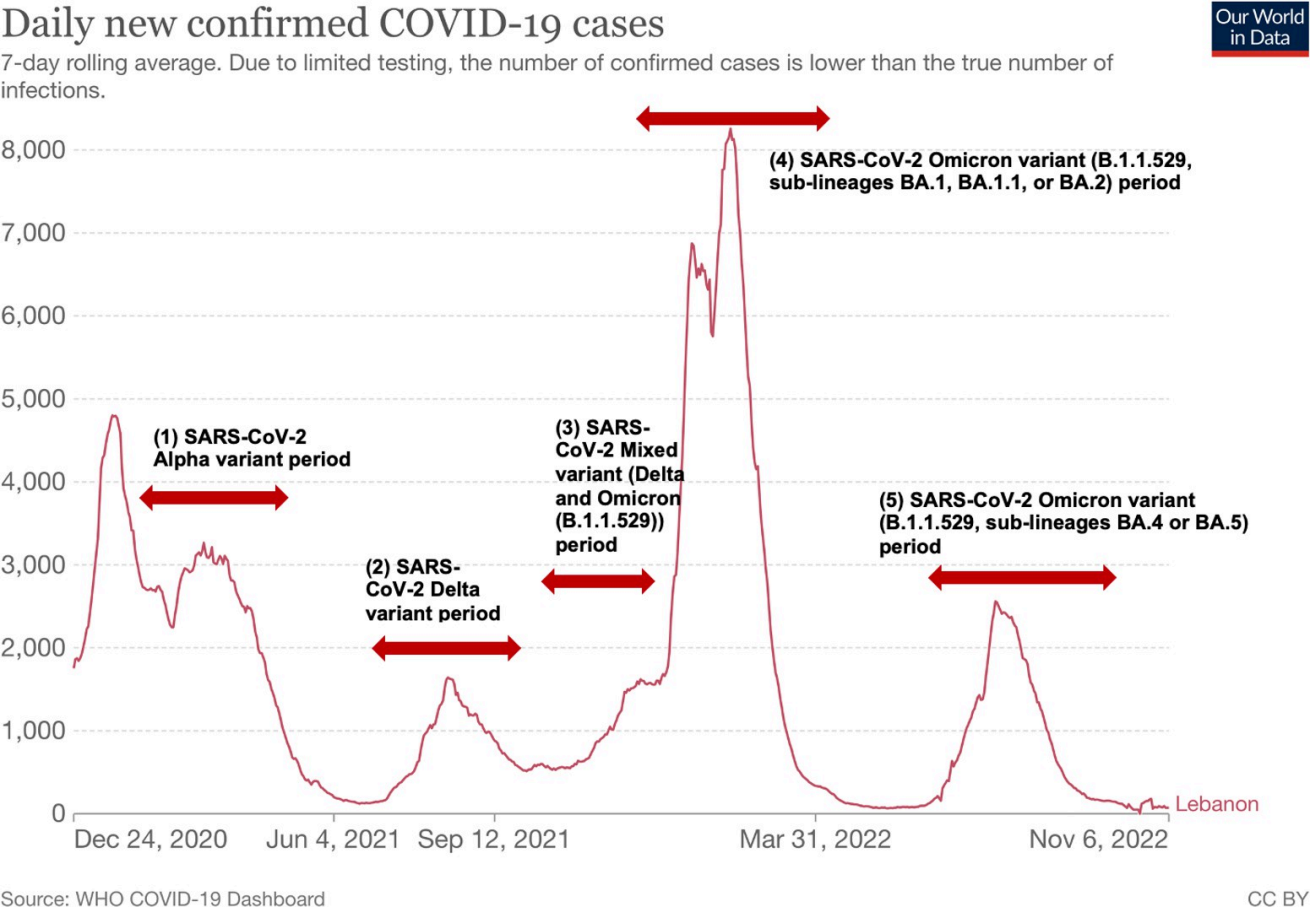
COVID-19 in Lebanon: The daily number of COVID-19 cases throughout the waves of the pandemic in Lebanon and associated SARS-CoV-2 variants between January 2021 and September 2022. Reference: Edouard Mathieu, Hannah Ritchie, Lucas Rodés-Guirao, Cameron Appel, Charlie Giattino, Joe Hasell, Bobbie Macdonald, Saloni Dattani, Diana Beltekian, Esteban Ortiz-Ospina and Max Roser (2020)—"Coronavirus Pandemic (COVID-19)". Published online at OurWorldInData.org. Retrieved from: ’https://ourworldindata.org/coronavirus’ [Online Resource]. N.B.: X-axis: month-year; Y-axis left: number of confirmed daily cases. In this study, we examined vaccine effectiveness during five periods of high or peak circulation of SARS-CoV-2 variants [21–24]: 1. **SARS-CoV-2 Alpha cases:** Personnel were categorized as such if they initially tested positive between February 21st, 2021, and May 31st, 2021, as the majority of cases in Lebanon were primarily attributable to the circulating Alpha variant during that period. 2. **SARS-CoV-2 Delta cases:** Personnel were classified as such if they first tested positive between July 21st, 2021, and November 7th, 2021, as the majority of cases in Lebanon were predominantly attributable to the circulating Delta variant during that time. 3. **SARS-CoV-2 Mixed (Delta and Omicron (B.1.1.529)) cases:** Personnel fell into this category if they first tested positive between November 8th, 2021, and December 31st, 2021. This period represented an overlap between two variants, with residual Delta infection incidence in the community alongside the gradual increase in Omicron infections. 4. **SARS-CoV-2 Omicron (B.1.1.529, sublineages BA.1, BA.1.1, or BA.2) cases:** Personnel were designated as such if they first tested positive between January 1st, 2022, and March 7th, 2022, as these sub-lineages predominantly circulated during that time frame. 5. **SARS-CoV-2 Omicron (B.1.1.529, sublineages BA.4 or BA.5) cases:** Personnel were identified as such if they first tested positive between June 21st, 2022, and September 7th, 2022, as these sub-lineages predominantly circulated during that period.

Coincidentally, the appearance of each variant and/or sub-lineage was followed by new surges in the number of cases in Lebanon [21–24]. We chose to study VE during five periods of high or peak circulation of SARS-CoV-2 variants, excluding data from the early and late stages of the wave. In the initial stages, transmission is slower, and toward the end of the wave, herd immunity begins to develop (Fig 1). Personnel who tested positive were only included up to September 8, 2022, as it marked the end of the study period. Both symptomatic and asymptomatic laboratory-confirmed infections were included. This approach ensured that the same individuals were followed over time, allowing for a comprehensive assessment of VE across multiple periods.

In each of the five periods, VE analyses were stratified according to primary immunization schemes (2× Sinopharm, 2× Pfizer, 2× Sputnik V, 2× AstraZeneca, or 1× Sinopharm/1× Pfizer). Cases and controls were categorized based on the elapsed time since the last vaccine dose. VE was assessed for each primary course in intervals of < 3 months, ≥ 3 –<6 months, and ≥6 months after the second dose. VE was assessed for the first booster (Sinopharm or Pfizer vaccine) in intervals of < 3 months, ≥ 3 –< 6 months, and ≥ 6 months after receiving the booster dose. VE was calculated for each vaccination scheme, including all vaccinees with and without previous exposure to COVID-19.

### 2.3 Humoral immunity

Between December 2022 and January 2023, we conducted a random prospective assessment of immunogenicity at various time points in vaccination subgroups that had previously received Sinopharm and Pfizer vaccines, and had not been exposed to COVID-19. Participants were categorized into different groups based on their primary vaccination type and whether they had received a booster dose (Table 1). The number of participants in each group during the immunogenicity assessment period was determined by the temporal definitions of the groups. Participants were further stratified by age and gender.

**Table 1:** Immunogenicity assessment groups based on the primary and booster vaccination schemes and timeframe from the second or booster dose.

**Type of COVID-19 Primary Vaccination Scheme**	**Timing of Blood Withdrawal After 2**^**nd**^ **COVID-19 Vaccine Dose**
2×SPh (21 days apart)	> 14 days and < 3 months
2×SPh	≥ 6 months
1×SPh/1×PFZ (21 days apart)	> 14 days and < 3 months
2×PFZ (21 days apart)	> 14 days and < 3 months
2×PFZ	≥ 3 months and < 6 months
2×PFZ	≥ 6 months
**Type of COVID-19 Booster Vaccination Scheme**	**Timing of Blood Withdrawal After 1**^**st**^ **Booster COVID-19 Vaccine Dose**
2×SPh/1×SPh (6 months apart)	> 7 days and < 3 months
2×SPh/1×PFZ (6 months apart)	> 7 days and < 3 months
2×PFZ/1×PFZ (6 months apart)	> 7 days and < 3 months
2×PFZ/1×SPh (6 months apart)	> 7 days and < 3 months

**Abbreviations:** SPh = Sinopharm (BBIBP-CorV), PFZ = Pfizer-BioNTech (BNT162b2).

#### 2.3.1 Blood sample collection and humoral immunity measurement

Blood samples were collected from participants in all groups at the Lebanese Army Military Hospital COVID-19 Vaccination Center and Makassed General Hospital COVID-19 Vaccination Center in Beirut. These samples were then sent to FMPS Holding–BIOTECK Laboratory for the determination of SARS-CoV-2 anti-S-IgG titers using immunoassay techniques. VIDAS®3 SARS-CoV-2 IgG (BioMérieux, France) assay was used to measure IgG against the RBD of the spike protein [25–27]. The assay principle is based on a 2-step enzyme immunoassay combined with an enzyme-linked fluorescent assay [25–27]. The test was conducted following the manufacturer’s instructions. Results were calculated as an index, representing the ratio between the relative fluorescence value (RFV) measured in the sample and the RFV obtained for the calibrator [25–27]. A result was considered negative when the index was <1.00 and positive when the index was ≥1.00 [25–27]. All readings were standardized to BAU/mL using the WHO international standard for the VIDAS®3 SARS-CoV-2 IgG (VIDAS®3 SARS-CoV-2 IgG index = 1 (cutoff) = 20.33 BAU/mL) [25–27].

### 2.4 Ethics approval

This study adhered to the Strengthening the Reporting of Observational Studies in Epidemiology reporting guidelines (S1 Table). It was conducted in accordance with the guidelines of the Declaration of Helsinki, and ethics approval was obtained from Makassed General Hospital’s Institutional Clinical Research Ethics Committee (approval numbers: 1112021 and 1102022). The section of the study related to VE was deemed not to involve human participants due to the retrospective nature of the study; thus, the requirement for informed consent was waived. During the data collection phase, only subject case numbers were included. At a later stage, a different number was assigned to each case to safeguard subject privacy. All methods were performed in accordance with the hospital’s institutional review board committee guidelines and regulations. However, participants in the immunogenicity subgroup analyses provided their written informed consent for the collection of their health information and blood samples, with a focus on ensuring anonymity.

### 2.5 Statistical analysis

Sociodemographic cohort characteristics, including age categories (<50 and ≥50–<65 years), gender (male and female), COVID-19 infection/recovery history before each variant surge (yes and no), personnel function (healthcare worker and non-healthcare worker), and the presence of comorbidities (none, one comorbidity or more), were compared between vaccination groups and presented as numbers and percentages. Categorical analyses were performed using the chi-square test.

For VE against infection during each of the five periods of high or peak circulation of SARS-CoV-2 variants, multivariable logistic regression was used, with laboratory-confirmed SARS-CoV-2 infections as the dependent variable and case participants being those who tested positive. Controls included personnel with no record of a positive SARS-CoV-2 PCR test during each period, either due to a lack of clinical suspicion or any possibility of contracting COVID-19 based on the contact tracing system implemented by the MoD since the start of the pandemic, irrespective of their vaccination status.

For VE against hospitalization during each of the five previously mentioned periods, multivariable logistic regression was used, with acute-care hospitalization due to laboratory-confirmed SARS-CoV-2 infections as the dependent variable, and case participants being those hospitalized. Controls included personnel with no record of acute-care hospitalization due to laboratory-confirmed SARS-CoV-2 infections during each period, as documented in the MoD healthcare database, irrespective of their vaccination status.

For VE against infection and hospitalization calculation, vaccination status was included as the independent variable, and the effectiveness of different vaccination schemes over the five time periods and the associated 95% confidence interval (CI) were calculated using the following equation: VE = [1 − adjusted odds ratio (aOR) of vaccination among cases compared with controls] × 100. VE was adjusted in logistic regression models for age categories, gender, COVID-19 infection/recovery history before each variant surge, personnel function, and the presence of comorbidities. These factors were all considered potential confounders and were included in all models. VE was calculated in the entire cohort, with the reference group for all estimates being the unvaccinated COVID-19-naïve personnel (i.e., individuals with no previous infection) in each of the five periods. Only those vaccinated in this specific time-since-vaccination stratum and those unvaccinated in each VE (infection) analysis for a specific time-since-vaccination stratum were included. Consequently, the number of cases and controls varied across the time-since-vaccination analyses.

Regarding immunogenicity subgroup analysis, categorical analyses on age and gender, presented as numbers and percentages, were performed using the chi-square test for different vaccination schemes at different time points among the selected participants. Antibody titers were presented as geometric mean titers (GMT) and 95% CIs. One-sample Kolmogorov–Smirnov test was used to check for data distribution normality. Kruskal–Wallis test, followed by Dunn’s multiple comparison post-hoc test, was performed to compare unpaired nonparametric data between the groups (antibody levels).

Statistical significance was defined as *p* < 0.05. The IBM Statistical Package for the Social Sciences program for Windows (version 23.0) (Armonk, NY, USA: IBM Corp.) and GraphPad Prism 9.0 software (GraphPad Software, Inc., San Diego, CA, USA) were used to conduct statistical analyses using two-tailed tests.

## 3. Results

### 3.1 Characteristics of the study population for VE analyses

**Fig 2:**
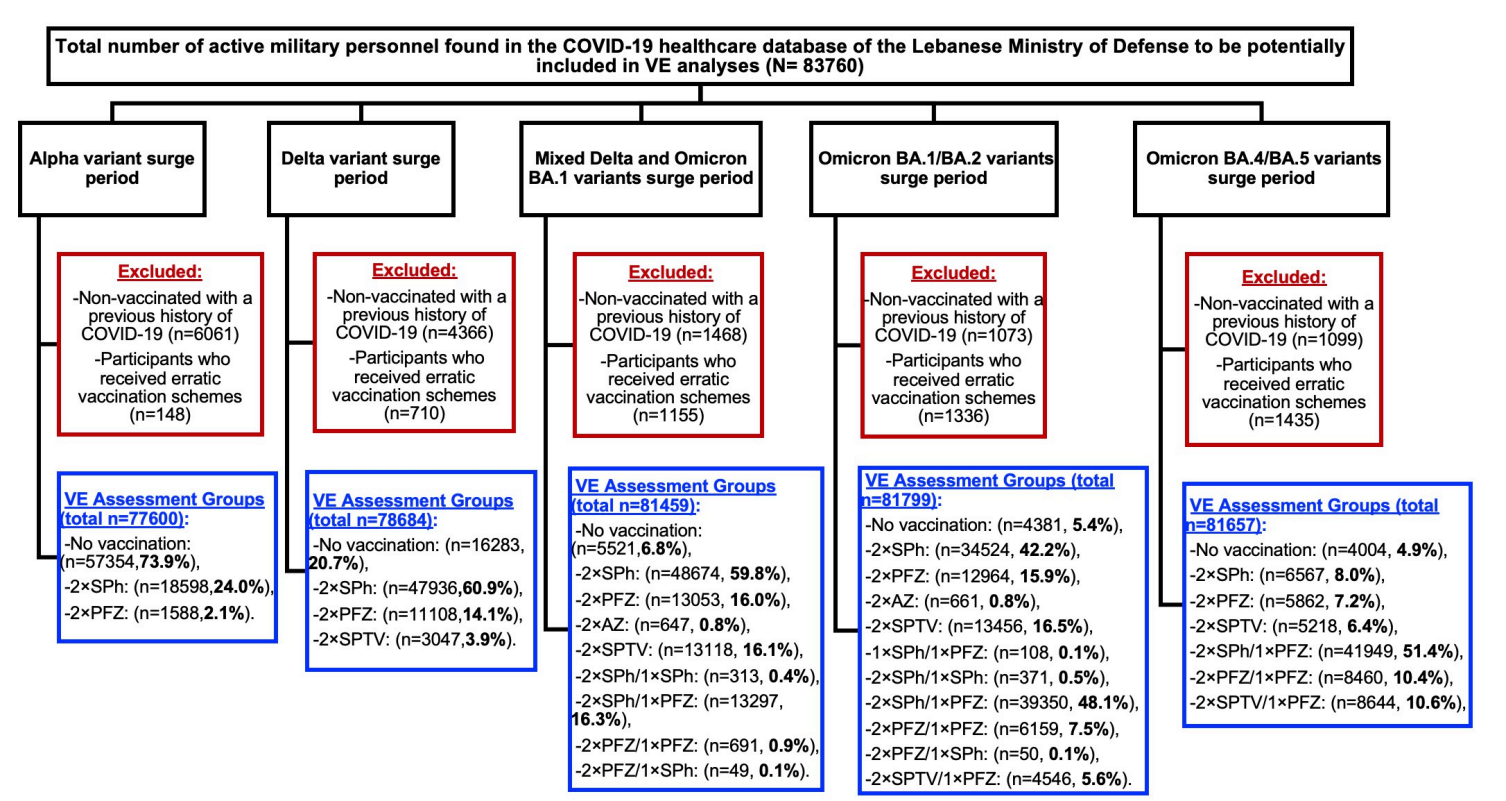
Study profile. Abbreviations: VE = vaccine effectiveness, SPh = Sinopharm (BBIBP-CorV), AZ = AstraZeneca (ChAdOx1 nCoV-19), PFZ = Pfizer-BioNTech (BNT162b2), SPTV = Sputnik V (Gam-COVID-Vac).

The total number of active military personnel identified in the COVID-19 healthcare database of the Lebanese MoD and longitudinally evaluated for VE across different time periods was 83,760. The distribution of vaccinees based on different vaccination schemes and non-vaccinated personnel during the study period is illustrated in Fig 2. Baseline demographics and characteristics of the study cohort according to the different vaccination combination groups are provided in S2 Table.

#### 3.1.1 Alpha variant surge period

During the period from February 21, 2021, to May 31, 2021, a total of 77,600 military personnel meeting the study inclusion criteria were included in the main analysis. The majority of participants were under 50 years of age (97.4%), with a median age of 32 years and an interquartile range of 27–38 years. In addition, 93.3% were males, 95.8% were non-healthcare workers, 4.2% were military healthcare workers, and 95.9% were healthy with no comorbidities.

#### 3.1.2 Delta variant surge period

Between July 21, 2021, and November 7, 2021, a total of 78,684 military personnel were included in the main analysis. The demographic distribution among different subgroups, including age, gender, function, and comorbidities, was comparable to that of the Alpha variant wave (S3 Table).

#### 3.1.3 “Mixed” (Delta and Omicron) variant surge period

Between November 8, 2021, and December 31, 2021, a total of 81,459 military personnel were included in the main analysis. Demographic characteristics among different subgroups remained consistent with those observed during previous waves (S4 Table).

#### 3.1.4 Omicron (B.1.1.529, sublineages BA.1, BA.1.1, or BA.2) variant surge period

Between January 1st, 2022, and March 7th, 2022, a total of 81,799 military personnel met the study inclusion criteria. Demographic characteristics among different subgroups remained consistent with those observed during previous waves (S5 Table).

#### 3.1.5 Omicron (B.1.1.529, sublineages BA.4 or BA.5) variant surge period

Between June 21st, 2022, and September 7th, 2022, a total of 81,657 military personnel met the study inclusion criteria. Demographic characteristics among different subgroups remained consistent with those observed during previous waves (S6 Table).

### 3.2 VE against COVID-19 infection across various periods and time points

#### 3.2.1 Alpha variant infection

**Fig 3:**
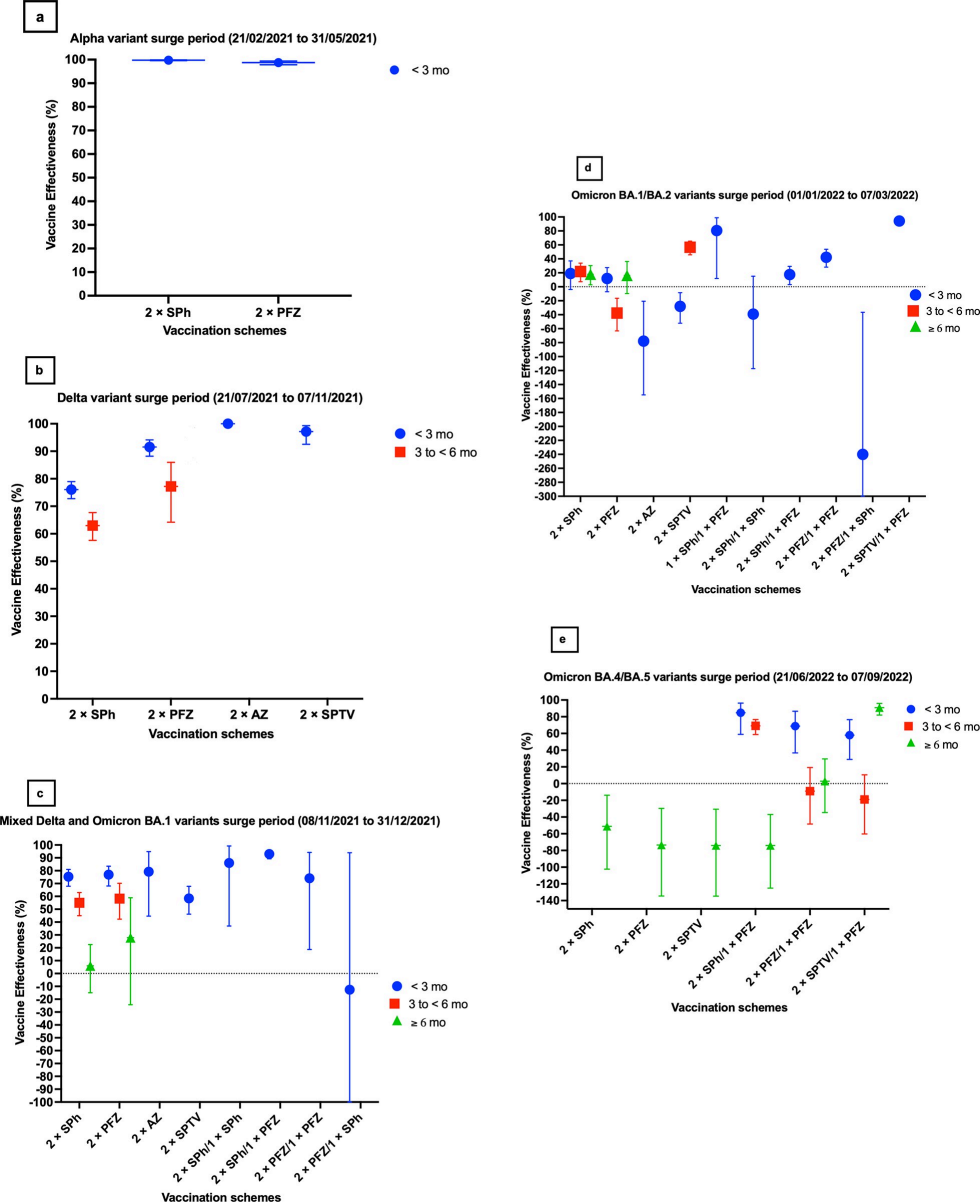
Effectiveness of the available COVID-19 vaccination schemes against laboratory-confirmed infections during the (A) Alpha variant wave, (B) Delta variant wave, (C) Mixed Delta and Omicron BA.1 variant wave,(D) BA.1/BA.2 Omicron variant wave, and (E) BA.4/BA.5 Omicron variant wave. Abbreviations: AZ = AstraZeneca (ChAdOx1 nCoV-19), mo = months, PFZ = Pfizer-BioNTech (BNT162b2), SPh = Sinopharm (BBIBP-CorV), SPTV = Gamaleya’s Sputnik V (Gam-COVID-Vac), VE = vaccine effectiveness. N.B. Data are presented as effectiveness point estimates, with error bars indicating the corresponding 95% confidence intervals.

*3*.*2*.*1*.*1 At less than 3 months after the second dose*. The effectiveness of Sinopharm primary vaccination scheme against infection was highest at 99.8% (95% CI, 99.6–99.9), similar to Pfizer primary vaccination scheme which reached 98.8% (95% CI, 97.84–99.36) (see Fig 3A and Table 2). No assessment of VE change with time against the Alpha variant infection was possible for any primary scheme during this wave.

**Table 2:** Effectiveness of the available COVID-19 primary and booster vaccination schemes against laboratory-confirmed infections during the different waves.

	Effectiveness against infection				
	Cases		Controls		Effectiveness (%) (95% confidence interval)
	Vaccinated	Unvaccinated	Vaccinated	Unvaccinated	
Alpha variant surge period (21/02/2021 to 31/05/2021)					
two-dose homologous priming vaccination regimens					
2 × SPh (< 3 mo)	12	12084	18586	45270	99.76 (99.6 to 99.87)
2 × PFZ (< 3 mo)	11	12084	1588	45270	98.75 (97.84 to 99.36)
Delta variant surge period (21/07/2021 to 07/11/2021)					
two-dose homologous priming vaccination regimens					
2 × SPh (< 3 mo)	410	658	47519	15625	76.06 (72.78 to 78.95)
2 × SPh (3 to < 6 mo)	342	658	25418	15625	62.98 (57.61 to 67.72)
2 × PFZ (< 3 mo)	38	658	9942	15625	91.54 (88.19 to 94.12)
2 × PFZ (3 to < 6 mo)	26	658	2415	15625	77.20 (64.20 to 85.98)
two-dose heterologous priming vaccination regimens					
2 × SPTV (< 3 mo)	3	658	3044	15625	97.14 (92.54 to 99.29)
Mixed Delta and Omicron BA.1 variants surge period (08/11/2021 to 31/12/2021)					
two-dose homologous priming vaccination regimens					
2 × SPh (< 3 mo)	110	127	19332	5394	75.29 (67.89 to 80.99)
2 × SPh (3 to < 6 mo)	481	127	44958	5394	55.02 (44.95 to 62.98)
2 × SPh (≥ 6 mo)	506	127	22979	5394	5.96 (−15.0 to 22.54)
2 × PFZ (< 3 mo)	55	127	10354	5394	76.9 (68.11 to 83.50)
2 × PFZ (3 to < 6 mo)	69	127	5949	5394	58.3 (42.26 to 70.21)
2 × PFZ (≥ 6 mo)	35	127	1736	5394	27.86 (−24.30 to 58.95)
2 × AZ (< 3 mo)	3	127	615	5394	79.18 (44.68 to 94.88)
two-dose heterologous priming vaccination regimens					
2 × SPTV (< 3 mo)	121	127	12903	5394	58.41 (46.19 to 67.84)
homologous boosting vaccination regimens					
2 × SPh/1 × SPh (< 3 mo)	1	127	310	5394	85.97 (36.96 to 99.20)
2 × PFZ/1 × PFZ (< 3 mo)	3	127	688	5394	74.11 (18.69 to 94.23)
heterologous boosting vaccination regimens					
2 × SPh/1 × PFZ (< 3 mo)	23	127	13273	5394	92.99 (89.23 to 95.64)
2 × PFZ/1 × SPh (< 3 mo)	1	127	48	5394	−12.6 (−498.2 to 93.94)
Omicron BA.1/BA.2 variants surge period (01/01/2022 to 07/03/2022)					
two-dose homologous priming vaccination regimens					
2 × SPh (< 3 mo)	99	196	2737	4185	18.92 (−3.80 to 37.02)
2 × SPh (3 to < 6 mo)	643	196	18015	4185	21.83 (7.15 to 33.88)
2 × SPh (≥ 6 mo)	631	196	18062	4185	17.9 (2.77 to 30.37)
2 × PFZ (< 3 mo)	257	196	5905	4185	11.86 (−7.20 to 27.45)
2 × PFZ (3 to < 6 mo)	614	196	9575	4185	−37.6 (−63.20 to −16.4)
2 × PFZ (≥ 6 mo)	132	196	2501	4185	16.2 (−9.80 to 36.27)
2 × AZ (< 3 mo)	36	196	448	4185	−77.8 (−154.90 to −20.70)
two-dose heterologous primary vaccination regimens					
2 × SPTV (< 3 mo)	695	196	12665	4185	−28.1 (−52.10 to −8.50)
2 × SPTV (3 to < 6 mo)	149	196	8132	4185	56.62 (45.79 to 65.33)
1 × SPh/1 × PFZ (< 3 mo)	1	196	115	4185	80.51 (11.82 to 98.90)
homologous boosting vaccination regimens					
2 × SPh/1 × SPh (< 3 mo)	21	196	346	4185	−39.2 (−117.2 to 15.15)
2 × PFZ/1 × PFZ (< 3 mo)	204	196	5945	4185	42.25 (28.08 to 53.69)
heterologous boosting vaccination regimens					
2 × SPh/1 × PFZ (< 3 mo)	1365	196	37936	4185	17.41 (3.17 to 29.18)
2 × PFZ/1 × SPh (< 3 mo)	8	196	41	4185	−240.0 (−666.0 to −36.5)
2 × SPTV/1 × PFZ (< 3 mo)	11	196	4532	4185	94.15 (89.72 to 97.01)
Omicron BA.4/BA.5 variants surge period (21/06/2022 to 07/09/2022)					
two-dose homologous priming vaccination regimens					
2 × SPh (≥ 6 mo)	165	68	6394	3936	−51.1 (−102.4 to −13.9)
2 × PFZ (≥ 6 mo)	174	68	5453	3936	−73.5 (−134.5 to −29.7)
two-dose heterologous primary vaccination regimens					
2 × SPTV (≥ 6 mo)	153	68	5061	3936	−74.1 (−134.6 to −30.6)
homologous boosting vaccination regimens					
2 × PFZ/1 × PFZ (< 3 mo)	8	68	1318	3936	68.76 (36.7 to 86.59)
2 × PFZ/1 × PFZ (3 to < 6 mo)	139	68	7410	3936	−9.1 (−48.4 to 19.19)
2 × PFZ/1 × PFZ (≥ 6 mo)	110	68	6269	3936	2.81 (−34.6 to 29.52)
heterologous boosting vaccination regimens					
2 × SPh/1 × PFZ (< 3 mo)	3	68	1115	3936	84.75 (58.9 to 96.27)
2 × SPh/1 × PFZ (3 to < 6 mo)	190	68	36771	3936	69.1 (58.72 to 76.59)
2 × SPh/1 × PFZ (≥ 6 mo)	1169	68	38833	3936	−74.1 (−125.1 to −37.0)
2 × SPTV/1 × PFZ (< 3 mo)	16	68	2222	3936	57.97 (29.0 to 76.57)
2 × SPTV/1 × PFZ (3 to < 6 mo)	164	68	8042	3936	−19.0 (−60.2 to 10.55)
2 × SPTV/1 × PFZ (≥ 6 mo)	8	68	5078	3936	90.82 (81.94 to 95.95)

Abbreviations: AZ = AstraZeneca (ChAdOx1 nCoV-19), mo = months, PFZ = Pfizer-BioNTech (BNT162b2), SPh = Sinopharm (BBIBP-CorV), SPTV = Gamaleya Sputnik V (Gam-COVID-Vac), VE = vaccine effectiveness.

#### 3.2.2 Delta variant infection

Overall, VE estimates for primary schemes during the Delta wave were lower than those reported during the Alpha wave (see Fig 3B and Table 2).

*3*.*2*.*2*.*1 At less than the first 3 months after the second dose*. Sinopharm’s VE against the Delta variant was 76.1% (95% CI, 72.8–79.0) at less than 3 months following the second dose, while Pfizer and Sputnik V primary regimens exhibited higher estimates, reaching 91.5% (95% CI, 88.2–94.1) and 97.1% (92.5–99.3), respectively (see Fig 3B and Table 2).

*3*.*2*.*2*.*2 Between 3 and less than 6 months after the second dose*. Effectiveness declined in primary schemes between 3 and less than 6 months after the second dose, with Sinopharm measuring 63% (57.6–67.7) and Pfizer measuring 77.2% (64.2–86.0) (see Fig 3B and Table 2). Changes in estimates of effectiveness against infection with the Delta variant could not be assessed for Sputnik V primary schemes during this timeframe.

There was no available data for VE at 6 months and beyond for any of the mentioned vaccination schemes against infection with the Delta variant.

#### 3.2.3 “Mixed” (Delta and Omicron) variant infection

Similar to the Delta variant phase, the effectiveness of primary schemes began to decline during this period compared with the Alpha variant wave and the period when the Delta variant was the dominant strain (see Fig 3C and Table 2).

*3*.*2*.*3*.*1 At less than 3 months after the second dose*. Sinopharm’s VE against “Mixed” variant infections was 75.3% (95% CI, 67.9–81.0) at less than 3 months after the second dose. The corresponding VE estimates for the Pfizer and AstraZeneca primary schemes were almost identical, measuring 76.9% (95% CI, 68.1–83.5) and 79.2% (95% CI, 44.7–94.9), respectively. Sputnik V exhibited the lowest effectiveness against infection, reaching 58.4% (95% CI, 46.2–67.8) (see Fig 3C and Table 2).

*3*.*2*.*3*.*2 Between 3 and less than 6 months after the second dose*. Between 3 and less than 6 months after the second dose, effectiveness similarly declined for Sinopharm and Pfizer primary schemes, measuring 55.0% (95% CI, 45.0–63.0) and 58.3% (95% CI, 42.3–70.2), respectively. (see Fig 3C and Table 2) Changes in VE against infection could not be assessed for Sputnik V and AstraZeneca primary schemes during this timeframe.

*3*.*2*.*3*.*3 At 6 months and beyond after the second dose*. At 6 months and beyond after the second dose, a sharp decline was observed in the effectiveness of Sinopharm, which reached 5.0% (95% CI, −15.0–22.5) (see Fig 3C and Table 2). The corresponding estimate for Pfizer was higher, measuring 27.9% (95% CI, −24.3–59.0). Changes in VE against infection could not be assessed for Sputnik V and AstraZeneca primary schemes during this timeframe.

*3*.*2*.*3*.*4 At less than 3 months after the booster dose*. Following the first homologous booster dose, VE rebounded to 86.0% (95% CI, 37.0–99.2) for 2× Sinopharm/1× Sinopharm regimen and to 74.1% (95% CI, 18.7–94.2) for 2× Pfizer/1× Pfizer regimen (see Fig 3C and Table 2). In contrast, following the first heterologous booster dose, VE rebounded significantly to 93.0% (95% CI, 89.2–95.6) for 2× Sinopharm/1× Pfizer regimen but did not result in an increase in VE estimates for 2× Pfizer/1× Sinopharm regimen, with a value of −12.6% (95% CI, −498.2–93.94) (see Fig 3C and Table 2). No data regarding boosting Sputnik V and AstraZeneca primary schemes were available during this timeframe. Changes in VE estimates against infection at different time points could not be assessed for booster schemes during this period.

#### 3.2.4 Omicron (B.1.1.529, sublineages BA.1, BA.1.1, or BA.2) variant infection

VE estimates showed a consistent decrease for the Omicron variant compared with the earlier variant waves across all post-vaccination timeframes and for all combinations of primary and first booster schemes investigated (see Fig 3D and Table 2).

*3*.*2*.*4*.*1 At less than 3 months after the second dose*. Sinopharm’s VE against Omicron variant infections was at its lowest, measuring 18.9% (95% CI, −3.8–37.0), at less than 3 months after the second dose (see Fig 3D and Table 2). Similarly, the VE estimate for Pfizer was also low, measuring 11.9% (95% CI, −7.2–27.5). Sputnik V and AstraZeneca primary schemes did not protect against infection, with VE estimates of −28.1% (95% CI, −52.1–−8.5) and −77.8% (95% CI, −154.9–−20.7), respectively. Interestingly, the VE estimate for the heterologous primary scheme, 1× Sinopharm/1× Pfizer, was surprisingly high, reaching 80.5% (95% CI, 11.8–98.9).

*3*.*2*.*4*.*2 Between 3 and less than 6 months after the second dose*. Between 3 and less than 6 months after the second dose, effectiveness remained consistently low for Sinopharm primary scheme, measuring 21.8% (95% CI, 7.2–33.9) (see Fig 3D and Table 2). The VE estimate of Pfizer primary scheme was consistently low for the previous time point, reaching negative values at −37.6% (95% CI, −63.20–−16.4). Similarly, the VE estimate of AstraZeneca primary scheme was negative, reaching −133.7% (95% CI, −228.4–−62.9). Interestingly, the VE estimate for Sputnik V primary scheme increased to 56.6% (95% CI, 45.8–65.3) (see Fig 3D and Table 2). There was no available data for the effectiveness of the heterologous primary scheme, 1× Sinopharm/1× Pfizer, at this time point.

*3*.*2*.*4*.*3 At 6 months and beyond after the second dose*. At 6 months and beyond after the second dose, effectiveness remained stable for Sinopharm for the previous time point, measuring 17.9% (95% CI, 2.8–30.4). (see Fig 3D and Table 2). The corresponding estimate for the Pfizer increased compared with that of the previous time point, reaching 16.2% (95% CI, −9.8–36.3) (see Fig 3D and Table 2). Changes in effectiveness could not be assessed for Sputnik V, AstraZeneca, and 1× Sinopharm/1× Pfizer primary schemes at this time point.

*3*.*2*.*4*.*4 At less than 3 months after the booster dose*. After receiving the first homologous booster dose, no protection against infection was provided in 2× Sinopharm/1× Sinopharm scheme, with values measuring −39.2% (95% CI, −117.2–15.2) (see Fig 3D and Table 2). However, the corresponding VE estimate for 2× Pfizer/1× Pfizer rebounded to 42.3% (95% CI, 28.1–53.7). Following the first heterologous booster dose, VE was low at 17.4% (95% CI, 3.17–29.18) for 2× Sinopharm/1× Pfizer regimen (see Fig 3D and Table 2). The VE estimates were negative in the case of 2× Pfizer/1× Sinopharm scheme, with a value of −240.0% (95% CI, −666.0–−36.5). Interestingly, the VE estimates for 2× Sputnik V/1× Pfizer were the highest, measuring 94.2% (95% CI, 89.7–97.0) (see Fig 3D and Table 2). No data were available regarding the boosting of AstraZeneca and the 1× Sinopharm/1× Pfizer primary schemes during this timeframe. Changes in VE estimates against infection with the Omicron variant at different time points could not be assessed for all booster schemes during this period.

#### 3.2.5 Omicron (B.1.1.529, sublineages BA.4 or BA.5) variant infection

VE estimates consistently decreased for the Omicron (B.1.1.529, sub-lineages BA.4 or BA.5) variant when compared with the earlier variant waves for the primary schemes investigated (see Fig 3E and Table 2). However, booster dose VE estimates showed variable results for the Omicron variant compared to earlier waves across all post-vaccination timeframes and booster schemes examined.

*3*.*2*.*5*.*1 At different time points after the second dose*. Data for VE of primary vaccination schemes were unavailable for timeframes less than 3 months after receiving the second dose and from 3 to less than 6 months after the second dose. At 6 months and beyond after the second dose, none of the primary schemes provided protection against infection (see Fig 3E and Table 2).

*3*.*2*.*5*.*2 At less than 3 months after the booster dose*. After receiving the first booster dose, the effectiveness of 2× Pfizer/1× Pfizer regimen rapidly rebounded to 68.8% (95% CI, 36.7–86.6) at less than 3 months after the booster dose. In contrast, the corresponding VE estimate for 2× Sinopharm/1× Pfizer regimen rebounded to 84.8% (95% CI, 58.9–96.3). Similarly, the VE estimates for 2× Sputnik V/1× Pfizer regimen rebounded to 58.0% (95% CI, 29.0–76.6) at less than 3 months after the booster dose (see Fig 3E and Table 2).

*3*.*2*.*5*.*3 Between 3 and less than 6 months after the booster dose*. Between 3 and less than 6 months after the booster dose, the effectiveness of 2× Pfizer/1× Pfizer regimen declined to −9.1% (95% CI, -48.4–19.2). The decline in VE estimates for 2× Sinopharm/1× Pfizer regimen was less steep, reaching 69.1% (95% CI, 58.7–76.6). Similarly, there was a sharp decline in the effectiveness of 2× Sputnik V/1× Pfizer regimen, measuring −19.0% (95% CI, −60.2–10.6) (see Fig 3E and Table 2).

*3*.*2*.*5*.*4 At 6 months and beyond after the booster dose*. At 6 months and beyond after the booster dose, the VE estimates of 2× Pfizer/1× Pfizer regimen remained low, measuring 2.81% (95% CI, -34.6–29.52). The corresponding VE estimates of 2× Sinopharm/1× Pfizer sharply declined to negative values, reaching −74.1% (95% CI, −125.1– −37.0). Interestingly, the VE estimates for the 2× Sputnik V/1× Pfizer regimen rebounded, reaching 90.8% (95% CI, 81.9–96.0) (see Fig 3E and Table 2).

### 3.3 VE against acute-care hospitalization due to COVID-19 infection across various periods and time points

#### 3.3.1 Alpha variant infection-related acute-care hospitalization

*3*.*3*.*1*.*1 At less than 3 months after the second dose*. Both the Sinopharm and Pfizer primary vaccination schemes demonstrated high effectiveness against hospitalization at less than 3 months following the second dose, with estimates of 98.6% (95% CI, 95.6–99.8) and 96.4% (95% CI, 83.0–99.8), respectively.

No significant change in effectiveness was observed for any primary scheme during this period.

**Fig 4:**
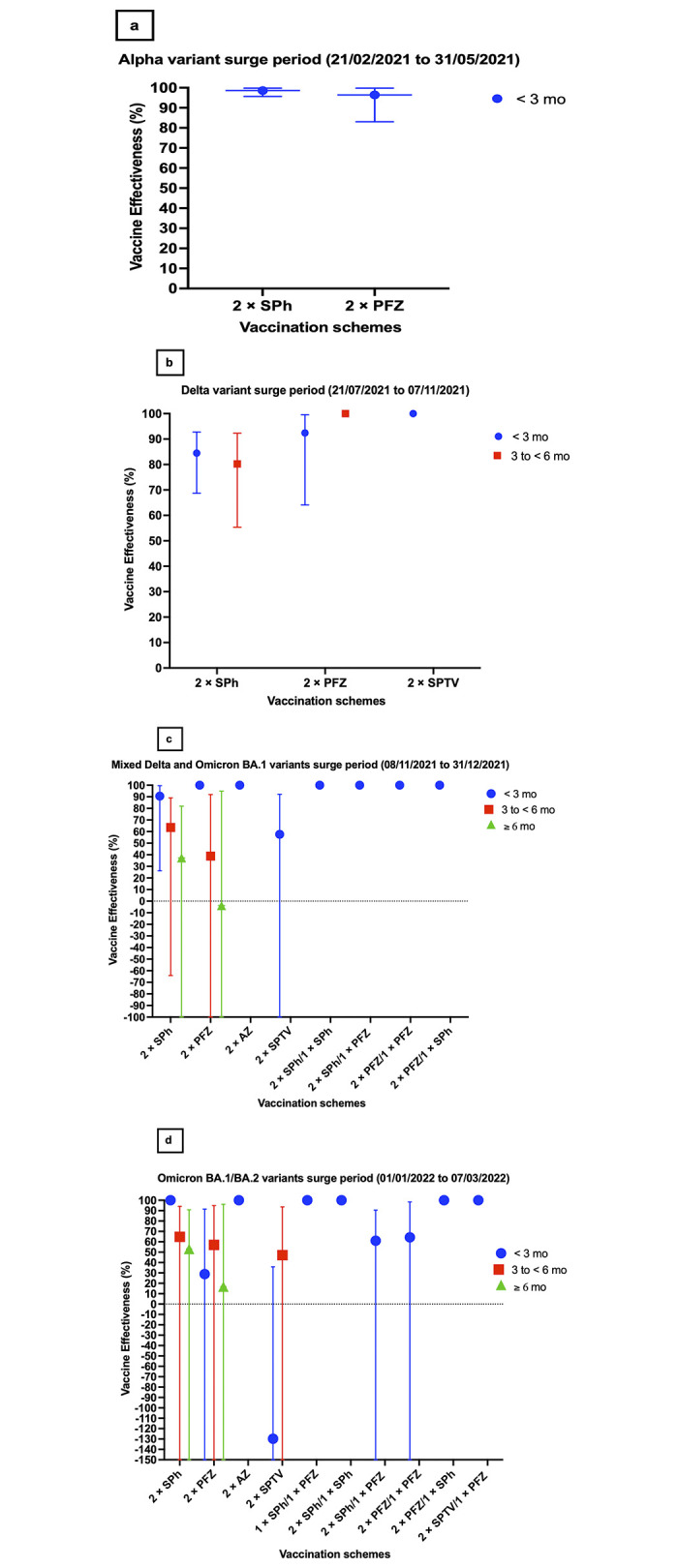
Effectiveness of the available COVID-19 vaccination schemes against acute-care hospitalization attributed to laboratory-confirmed infections during the (A) Alpha variant wave, (B) Delta variant wave, (C) Mixed Delta and Omicron BA.1 variant wave, and (D) BA.1/BA.2 Omicron variant wave. Abbreviations: AZ = AstraZeneca (ChAdOx1 nCoV-19), mo = months, PFZ = Pfizer-BioNTech (BNT162b2), SPh = Sinopharm (BBIBP-CorV), SPTV = Gamaleya’s Sputnik V (Gam-COVID-Vac), VE = vaccine effectiveness. N.B. Data are presented as effectiveness point estimates, with error bars indicating the corresponding 95% confidence intervals.

Additionally, no critical or fatal COVID-19 cases were reported among breakthrough infections. (see Fig 4A and Table 3).

**Table 3:** Effectiveness of the available COVID-19 primary and booster vaccination schemes against acute-care hospitalization attributed to laboratory-confirmed infections during the different waves.

	Effectiveness against acute-care hospitalization				
	Cases		Controls		Effectiveness % (95% confidence interval)
	Vaccinated	Unvaccinated	Vaccinated	Unvaccinated	
Alpha variant surge period (21/02/2021 to 31/05/2021)					
two-dose homologous priming vaccination regimens					
2 × SPh (< 3 mo)	2	411	18596	56943	98.59 (95.62 to 99.77)
2 × PFZ (< 3 mo)	1	411	1598	56943	96.40 (83.0 to 99.80)
Delta variant surge period (21/07/2021 to 07/11/2021)					
two-dose homologous priming vaccination regimens					
2 × SPh (< 3 mo)	11	23	47918	16260	84.47 (68.71 to 92.76)
2 × SPh (3 to < 6 mo)	8	23	25752	16260	80.22 (55.35 to 92.30)
2 × PFZ (< 3 mo)	1	23	9979	16260	92.42 (64.07 to 99.58)
2 × PFZ (3 to < 6 mo)	0	23	2441	16260	100
two-dose heterologous priming vaccination regimens					
2 × SPTV (< 3 mo)	0	23	3047	16260	100
Mixed Delta and Omicron BA.1 variants surge period (08/11/2021 TO 31/12/2021)					
two-dose homologous priming vaccination regimens					
2 × SPh (< 3 mo)	1	3	19441	5518	90.54 (26.12 to 99.53)
2 × SPh (3 to < 6 mo)	9	3	45430	5518	63.56 (−64.2 to 89.13)
2 × SPh (≥ 6 mo)	8	3	23477	5518	37.32 (−186.2 to 81.88)
2 × PFZ (< 3 mo)	0	3	10409	5518	100
2 × PFZ (3 to < 6 mo)	2	3	6016	5518	38.85 (−269.1 to 91.95)
2 × PFZ (≥ 6 mo)	1	3	1770	5518	−3.90 (−711.9 to 94.86)
2 × AZ (< 3 mo)	0	3	615	5518	100
two-dose heterologous priming vaccination regimens					
2 × SPTV (< 3 mo)	3	3	13021	5518	57.62 (−129 to 92.16)
homologous boosting vaccination regimens					
2 × SPh/1 × SPh (< 3 mo)	0	3	311	5518	100
2 × PFZ/1 × PFZ (< 3 mo)	0	3	691	5518	100
heterologous boosting vaccination regimens					
2 × SPh/1 × PFZ (< 3 mo)	0	3	13296	5518	100
2 × PFZ/1 × SPh (< 3 mo)	0	3	48	5518	100
Omicron BA.1/BA.2 variants surge period (01/01/2022 to 07/03/2022)					
two-dose homologous priming vaccination regimens					
2 × SPh (< 3 mo)	0	2	2836	4379	100
2 × SPh (3 to < 6 mo)	3	2	18655	4379	64.79 (−167.3 to 94.17)
2 × SPh (≥ 6 mo)	4	2	18689	4379	53.14 (−238 to 90.86)
2 × PFZ (< 3 mo)	2	2	6160	4379	28.91 (−492.4 to 91.47)
2 × PFZ (3 to < 6 mo)	2	2	10187	4379	57.01 (−258.2 to 94.84)
2 × PFZ (≥ 6 mo)	1	2	2632	4379	16.81 (−768.6 to 96.13)
2 × AZ (< 3 mo)	0	2	484	4379	100
two-dose heterologous primary vaccination regimens					
2 × SPTV (< 3 mo)	14	2	13346	4379	−129.7 (−1363 to 35.86)
2 × SPTV (3 to < 6 mo)	2	2	8279	4379	47.11 (−340.7 to 93.65)
1 × SPh/1 × PFZ (< 3 mo)	0	2	115	4379	100
homologous boosting vaccination regimens					
2 × SPh/1 × SPh (< 3 mo)	0	2	367	4379	100
2 × PFZ/1 × PFZ (< 3 mo)	1	2	6148	4379	64.31 (−272.6 to 98.34)
heterologous boosting vaccination regimens					
2 × SPh/1 × PFZ (< 3 mo)	7	2	39294	4379	61.0 (−161.8 to 90.58)
2 × PFZ/1 × SPh (< 3 mo)	0	2	41	4379	100
2 × SPTV/1 × PFZ (< 3 mo)	0	2	4543	4379	100
Omicron BA.4/BA.5 variants surge period (21/06/2022 to 07/09/2022)					
two-dose homologous priming vaccination regimens					
2 × SPh (≥ 6 mo)	0	1	6559	4003	100
2 × PFZ (≥ 6 mo)	0	1	5627	4003	100
two-dose heterologous primary vaccination regimens					
2 × SPTV (≥ 6 mo)	0	1	5214	4003	100
homologous boosting vaccination regimens					
2 × PFZ/1 × PFZ (< 3 mo)	0	1	1326	4003	100
2 × PFZ/1 × PFZ (3 to < 6 mo)	0	1	7549	4003	100
2 × PFZ/1 × PFZ (≥ 6 mo)	1	1	6378	4003	37.2 (−270.5 to 97.5)
heterologous boosting vaccination regimens					
2 × SPh/1 × PFZ (< 3 mo)	0	1	1118	4003	100
2 × SPh/1 × PFZ (3 to < 6 mo)	0	1	36961	4003	100
2 × SPh/1 × PFZ (≥ 6 mo)	0	1	40002	4003	100
2 × SPTV/1 × PFZ (< 3 mo)	0	1	2238	4003	100
2 × SPTV/1 × PFZ (3 to < 6 mo)	1	1	8205	4003	51.2 (-268.3 to 98.1)
2 × SPTV/1 × PFZ (≥ 6 mo)	0	1	5086	4003	100

**Abbreviations:** AZ = AstraZeneca (ChAdOx1 nCoV-19), mo = months, PFZ = Pfizer-BioNTech (BNT162b2), SPh = Sinopharm (BBIBP-CorV), SPTV = Gamaleya Sputnik V (Gam-COVID-Vac), VE = vaccine effectiveness.

#### 3.3.2 Delta variant infection-related acute-care hospitalization

Overall, VE estimates against hospitalization for primary schemes during the Delta wave were lower than those reported during the Alpha wave (see Fig 4B and Table 3).

*3*.*3*.*2*.*1 At less than 3 months after the second dose*. Sinopharm’s effectiveness was highest at 84.5% (95% CI, 68.7–92.8) at less than 3 months following the second dose, while Pfizer’s estimate was higher at 92.4% (95% CI, 64.1–99.6). No acute-care hospitalizations were reported for Sputnik V primary regimen during this timeframe. (see Fig 4B and Table 3).

*3*.*3*.*2*.*2 Between 3 and less than 6 months after the second dose*. Sinopharm’s effectiveness slightly declined to 80.2% (95% CI, 55.4–92.3) between 3 and less than 6 months after the second dose. No acute-care hospitalizations were reported for Pfizer primary regimen during this timeframe. Changes in the VE estimates could not be assessed for Sputnik V primary scheme during this timeframe.

There was no available data for VE against hospitalization at 6 months and beyond for any of the mentioned vaccination schemes.

None of the breakthrough infections in any of the vaccination groups progressed to critical or fatal COVID-19 during this period. (see Fig 4B and Table 3).

#### 3.3.3 “Mixed” (Delta and Omicron) variant infection-related acute-care hospitalization

*3*.*3*.*3*.*1 At less than 3 months after the second dose*. Sinopharm’s effectiveness against “Mixed” variant infections-related acute-care hospitalization was 90.5% (95% CI, 26.1–99.5) at less than 3 months after the second dose (see Fig 4C and Table 3). No acute-care hospitalizations were reported for Pfizer and AstraZeneca primary regimens during this timeframe. Sputnik V’s effectiveness against acute-care hospitalization was the lowest among all mentioned primary schemes, reaching 57.6% (95% CI, −129.0–92.2) (see Fig 4C and Table 3).

*3*.*3*.*3*.*2 Between 3 and less than 6 months after the second dose*. Between 3 and less than 6 months after the second dose, the VE estimate against hospitalization declined for Sinopharm and Pfizer primary schemes, measuring 63.6% (95% CI, −64.2–89.1) and 38.9% (95% CI, −269.1–92.0), respectively. (see Fig 4C and Table 3). Changes in VE against hospitalization could not be assessed for Sputnik V and AstraZeneca primary schemes during this period.

*3*.*3*.*3*.*3 At 6 months and beyond after the second dose*. At 6 months and beyond after the second dose, effectiveness further declined for Sinopharm primary schemes, measuring 37.3% (95% CI, −186.2–81.9). (see Fig 4C and Table 3). A steep decline was reported for Pfizer primary schemes against hospitalization, measuring −3.9% (95% CI, −711.9–94.9) (see Fig 4C and Table 3). Changes in VE against hospitalization could not be assessed for Sputnik V and AstraZeneca primary schemes during this period.

*3*.*3*.*3*.*4 At less than 3 months after the booster dose*. Following the first homologous or heterologous booster dose in Sinopharm and Pfizer primary schemes, no breakthrough infections were reported in any group less than 3 months after the third dose (see Fig 4C and Table 3). No data was available regarding the boosting of Sputnik V and AstraZeneca primary schemes during this period.

Changes in the effectiveness estimates at different time points could not be assessed for booster schemes during this period.

None of the breakthrough infections in any of the primary or booster vaccination groups progressed to critical or fatal COVID-19 during this period.

#### 3.3.4 Omicron (B.1.1.529, sublineages BA.1, BA.1.1, or BA.2) variant infection-related acute-care hospitalization

*3*.*3*.*4*.*1 At less than 3 months after the second dose*. No acute-care hospitalizations were reported for the Sinopharm, AstraZeneca, or 1× Sinopharm/1× Pfizer primary regimens at less than 3 months after the second dose. Pfizer’s effectiveness was 28.9% (95% CI, −492.4–91.5) during the same time point. However, Sputnik V’s effectiveness against hospitalization was the lowest among all mentioned primary schemes, measuring −129.7% (95% CI, −1363–35.86) (see Fig 4D and Table 3).

*3*.*3*.*4*.*2 Between 3 and less than 6 months after the second dose*. Between 3 and less than 6 months after the second dose, effectiveness declined for the Sinopharm primary scheme, measuring 64.8% (95% CI, −167.3–94.2) (see Fig 4D and Table 3). However, it was not the case for Pfizer and Sputnik V where VE estimates were unexpectedly higher than that of the previous time point, reaching 57.01% (95% CI, −258.2–94.84) and 47.11% (95% CI, −340.7–93.65), respectively (see Fig 4D and Table 3).

*3*.*3*.*4*.*3 At 6 months and beyond after the second dose*. At 6 months and beyond after the second dose, effectiveness slightly declined for the Sinopharm primary schemes for the previous time point, measuring 53.1% (95% CI, −238–90.86) (see Fig 4D and Table 3). A decline was reported for the Pfizer primary schemes, measuring 16.81% (95% CI, −768.6–96.13) (see Fig 4D and Table 3).

*3*.*3*.*4*.*4 At less than 3 months after the booster dose*. After receiving the first booster dose, effectiveness against hospitalization rebounded and was reported to be similar between 2× Sinopharm/1× Pfizer and 2× Pfizer/1× Pfizer, measuring 61.0% (95% CI, −161.8–90.6) and 64.3% (95% CI, −272.6–98.3), respectively, at less than 3 months after the booster dose (see Fig 4D and Table 3).

No acute-care hospitalizations were reported for 2× Sinopharm/1× Sinopharm, 2× Pfizer/1× Sinopharm, and 2× Sputnik V/1× Pfizer regimens at less than 3 months after the third dose. No data were available regarding boosting the AstraZeneca primary scheme during this period.

Changes in the VE estimates at different time points could not be assessed for booster schemes during this period.

None of the breakthrough infections in any of the primary or booster vaccination groups progressed to critical or fatal COVID-19 during this period.

#### 3.3.5 VE against omicron (B.1.1.529, Sublineages BA.4 or BA.5) variant infection-related acute-care hospitalization

*3*.*3*.*5*.*1 Across different time points after the second dose*. No acute-care hospitalizations were reported across all post-vaccination timeframes for the investigated primary schemes (Table 3).

*3*.*3*.*5*.*2 At less than 3 months after the booster dose*. No acute-care hospitalizations were reported across all combinations for the investigated booster schemes (Table 3).

*3*.*3*.*5*.*3 Between 3 and less than 6 months after the booster dose*. No acute-care hospitalizations were reported for 2×Sinopharm/1× Pfizer and 2× Pfizer/1× Pfizer regimens between 3 and less than 6 months after the third dose (Table 3). However, the VE estimate of the 2× Sputnik V/1× Pfizer regimen measured 51.2% (95% CI, −268.3–98.1).

*3*.*3*.*5*.*4 At 6 months and beyond after the booster dose*. No acute-care hospitalizations were reported for 2× Sinopharm/1× Pfizer and 2× Sputnik V/1× Pfizer regimens at 6 months and beyond after the booster dose (Table 3). However, the VE estimate of the 2× Pfizer/1× Pfizer regimen measured 37.2% (95% CI, −270.5–97.5).

None of the breakthrough infections in any of the primary or booster vaccination groups progressed to critical or fatal COVID-19 during this period.

### 3.4 Assessment of humoral immunity of different vaccination schemes in the subgroup analysis

**Fig 5:**
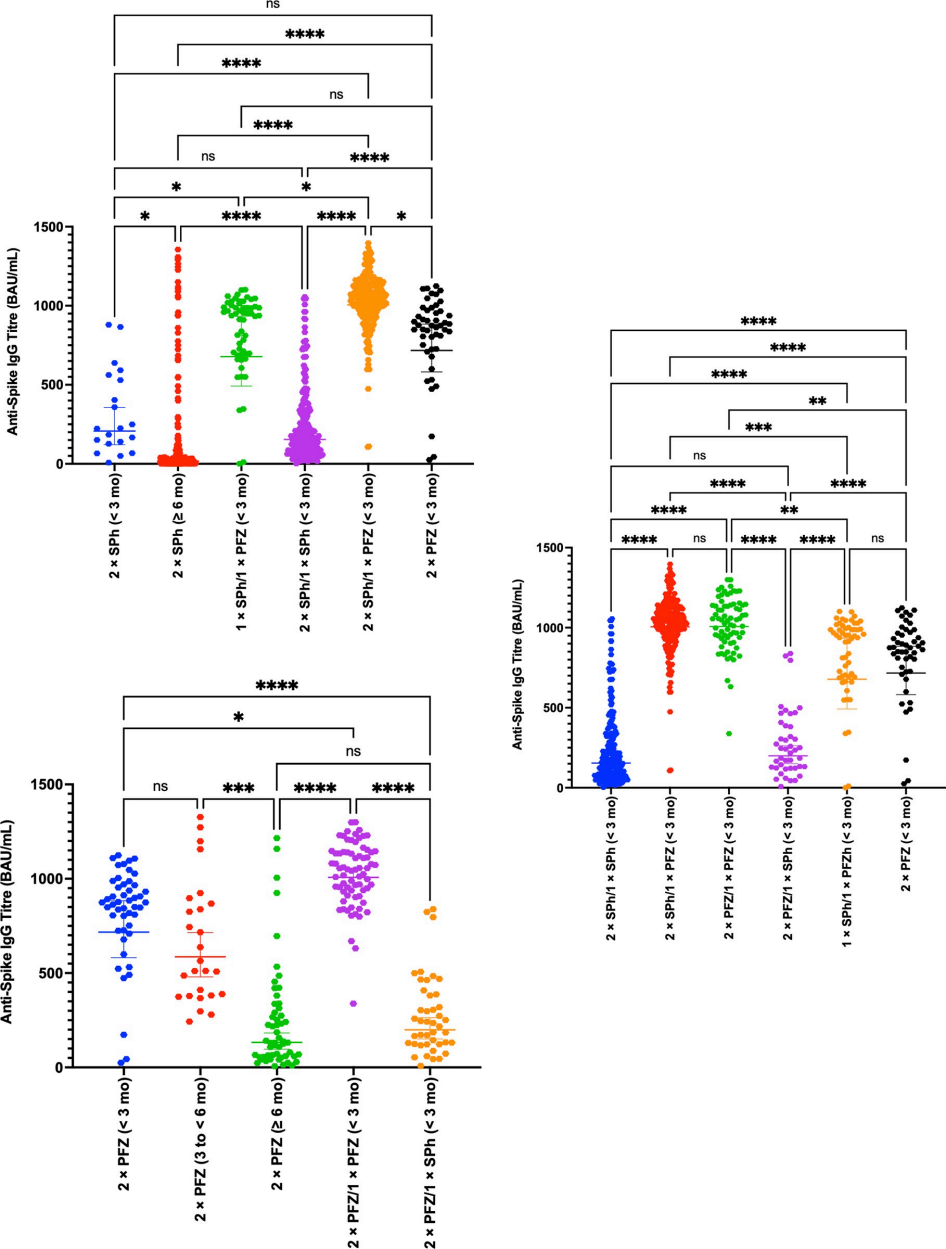
Humoral immune responses to SARS-CoV-2 spike protein following various primary vaccination schemes and booster doses with Sinopharm or Pfizer vaccines among COVID-19-naïve participants at different time points. Abbreviations: IgG = immunoglobulin G, BAU = Binding Antibody Unit, mo = months, PFZ = Pfizer-BioNTech (BNT162b2), SPh = Sinopharm (BBIBP-CorV). N.B: The initial measurement for anti-S-IgG was deemed negative if the index was < 1.00 (seronegative) and positive if the index was ≥ 1.00 (seropositive). All readings were standardized to BAU/mL using the WHO international standard for the VIDAS®3 SARS-CoV-2 IgG (VIDAS®3 SARS-CoV-2 IgG index = 1 (cutoff) = 20.33 BAU/mL). Antibody titers were reported as geometric mean titers (GMT) with corresponding 95% confidence intervals (CIs). One-sample Kolmogorov–Smirnov test was used to assess data distribution normality. Kruskal–Wallis test, followed by Dunn’s multiple comparison post-hoc test, was used to compare unpaired nonparametric data among the groups (antibody levels). ns (not significant), *P* > 0.05, *: *P* ≤ 0.05, ***: *P* ≤ 0.001, ****: *P* ≤ 0.0001.

The majority of participants included in this subgroup analysis, who received various vaccination schemes, were predominantly males under 50 years of age and had no comorbidities. Demographic characteristics of the participants recruited for the immunogenicity analysis are presented in S7 Table. Results of the anti-S-IgG GMT (BAU/ml) in the groups are presented in Fig 5A–5C and Table 4.

**Table 4:** Immunogenicity results of different vaccination schemes at different time points.

Vaccination Groups	Number of participants	Seropositive	Seronegative	Geometric mean titre of Anti-S RBD IgG (BAU/ml) (95% Confidence Interval)
SPh primary vaccination scheme				
2 × SPh (< 3 mo)	20	15 (93.75%)	1 (6.25%)	207.3 (120.8–355.8)
2 × SPh (≥ 6 mo)	228	105 (46.1%)	123 (53.9%)	16.5 (12.1–22.4)
1 × SPh/1 × PFZ (< 3 mo)	56	54 (96.4%)	2 (3.6%)	677.7 (491.9–933.7)
Booster dose after SPh primary vaccination scheme				
2 × SPh/1 × SPh (< 3 mo)	222	217 (97.8%)	5 (2.2%)	154.4 (134.9–176.8)
2 × SPh/1 × PFZ (< 3 mo)	228	228 (100%)	0	1005 (971.6–1040)
PFZ primary vaccination scheme				
2 × PFZ (< 3 mo)	48	48 (100%)	0	716.8 (581.5–883.5)
2 × PFZ (3 to < 6 mo)	26	26 (100%)	0	585.9 (479.9–715.4)
2 × PFZ (≥ 6 mo)	55	52 (94.6%)	3 (5.4%)	132.5 (96.2–182.4)
Booster dose after PFZ primary vaccination scheme				
2 × PFZ/1 × PFZ (< 3 mo)	55	55 (100%)	0	1007 (960.9–1055)
2 × PFZ/1 × SPh (< 3 mo)	43	42 (97.7%)	1 (2.3%)	199.2 (150.6–263.3)

Abbreviations: Anti-S RBD IgG = antispike receptor binding domain immunoglobulin G, BAU = Binding Antibody Unit, mo = months, PFZ = Pfizer-BioNTech (BNT162b2), SPh = Sinopharm (BBIBP-CorV).

N.B

- The initial measurement for anti-S-IgG was deemed negative if the index was < 1.00 (seronegative) and positive if the index was ≥ 1.00 (seropositive).
- All readings were standardized to BAU/mL using the WHO international standard for the VIDAS®3 SARS-CoV-2 IgG (VIDAS®3 SARS-CoV-2 IgG index = 1 (cutoff) = 20.33 BAU/mL).

#### 3.4.1 Sinopharm’s primary vaccination scheme

Anti-S-IgG GMT was 207.3 BAU/ml (95% CI, 120.8–355.8) at less than 3 months after the second Sinopharm dose among COVID-19-naïve participants (see Fig 5A and Table 4). This value significantly decreased to reach 16.5 BAU/ml (95% CI, 12.1–22.4) 6 months after the second dose or thereafter (*p* = 0.04).

#### 3.4.2 Pfizer’s primary vaccination scheme

Anti-S-IgG GMT was 716.8 BAU/ml (95% CI, 581.5–883.5) at less than 3 months after the second Pfizer dose among COVID-19-naïve participants (see Fig 5B and Table 4). This value decreased to reach 132.5 BAU/ml (95% CI, 96.2–182.4) 6 months after the second dose or thereafter (*p* < 0.0001).

Notably, anti-S-IgG GMT achieved by 2× Pfizer was significantly higher than that achieved by 2× Sinopharm (*p* < 0.0001) at less than 3 months after the second dose.

#### 3.4.3 1× Sinopharm/1× Pfizer primary vaccination scheme

Among participants who received 1× Sinopharm/1× Pfizer as a heterologous primary vaccination scheme, anti-S-IgG GMT measured highest at 677.7 BAU/ml (95% CI, 491.9–933.7) at less than 3 months of the second dose (see Fig 5A and Table 4). For comparison with Sinopharm primary scheme, this titer was significantly higher than that achieved after 2× Sinopharm (*p* = 0.03) at the same timeframe from the last dose. In addition, anti-S-IgG GMT achieved by 1× Sinopharm/1× Pfizer was almost comparable to that achieved by 2× Pfizer (*p* > 0.99) at less than 3 months after the second dose (see Fig 5C and Table 4).

#### 3.4.4 Pfizer’s booster vaccination scheme

After receiving the Pfizer booster dose, the anti-S-IgG GMT significantly rebounded to 1005 BAU/ml (95% CI, 971.6–1040) for 2× Sinopharm/1× Pfizer (p < 0.0001) and to 1007 BAU/ml (95% CI, 960.9–1055) for 2× Pfizer/1× Pfizer (p = 0.01) at less than 3 months after the booster dose (see Fig 5A and 5B and Table 4). Anti-S-IgG GMT achieved by 2× Pfizer/1× Pfizer and 2× Sinopharm/1× Pfizer were identical (p > 0.99) (see Fig 5C and Table 4). Notably, the anti-S-IgG GMT among participants who received 1× Sinopharm/1× Pfizer as a heterologous primary vaccination scheme was significantly lower than that achieved after 2× Sinopharm/1× Pfizer (p = 0.01) and 2× Pfizer/1× Pfizer (p < 0.0001).

#### 3.4.5 Sinopharm’s booster vaccination scheme

Following Sinopharm booster dose at less than 3 months, the anti-S-IgG GMT measured 154.4 BAU/ml (95% CI, 134.9–176.8) for individuals who received 2× Sinopharm/1× Sinopharm, showing no significant increase compared to Sinopharm primary scheme titers (*p* > 0.99) (see Fig 5A and Table 4). After receiving a Sinopharm booster dose at less than 3 months, anti-S-IgG GMT measured 199.2 BAU/ml (95% CI, 150.6–263.3) for individuals who received 2× Pfizer/1× Sinopharm, demonstrating a significant decrease compared to the titers achieved with the 2× Pfizer scheme at the same timeframe (*p* < 0.0001) (see Fig 5B and Table 4).

## 4. Discussion

### 4.1 Study scope and cohort characteristics

After 4 years of the COVID-19 pandemic, we have gained a comprehensive understanding of vaccine dynamics [17, 28–30]. Our study provided a unique opportunity to compare the effectiveness of different vaccines administered to the same population simultaneously, a scenario that is uncommon in many settings. Typically, countries rely on a single vaccine type or platform during specific periods as part of their national vaccination programs. For instance, the US and most European countries predominantly used mRNA vaccines, while China relied on inactivated vaccines [17, 28–30].

In contrast to controlled trials and laboratory studies that assess immunogenicity or efficacy, which are conducted in controlled environments with specific timeframes, subjects, and locations, VE measurement relies on real-world data [13, 31]. Real-world effectiveness of vaccines is influenced by numerous factors, including individual immune responses, adherence to vaccination schedules, and community-level influences [13, 31]. In addition, VE can vary even for the same vaccine within the same population, depending on when the effectiveness was assessed during a variant wave [19]. As previously mentioned, at the time of the outbreak, transmission is slower, and toward the end of the wave, herd immunity builds up, potentially leading to a false negative impact on calculated VE. This effect could mask the true effectiveness of the vaccine when calculated. Given these considerations, we chose to focus solely on the peak of the variant wave, excluding data from the early and late stages of the wave.

Our study cohort exhibited relatively homogenous demographic and other characteristics. It primarily comprised young, healthy, nonhealthcare worker males, with a median age of 32 years (interquartile range, 27–38 years). Vaccine responsiveness and severity of breakthrough infections are influenced by factors such as age, immune conditions, and comorbidities [32]. To minimize the impact of these confounding variables in more diverse populations, we selected a relatively homogeneous cohort of generally healthy individuals for immunogenicity and effectiveness studies. However, the effects of the vaccine on patients with different clinical characteristics should be explored in separate, well-defined cohorts. This approach offers a more comprehensive perspective by examining various facets of VE and mitigating the influence of confounding factors.

### 4.2 VE against documented infection across the different vaccination schemes and variants

In this case-control study, the effectiveness of primary COVID-19 vaccine schemes, including Sinopharm, Pfizer, and Sputnik V, remained consistently high during the Alpha and Delta waves, despite a decline in the latter. However, during the Omicron wave, significant declines in VE against infection were observed across all vaccine platforms. This decline began during the phase when “Mixed” Delta and Omicron variants were present between November and December 2021 and persisted until the Omicron variants predominated in 2022. Primary vaccination schemes involving Sinopharm, Pfizer, Sputnik V, and AstraZeneca lost their protective effect against infection. Nonetheless, a heterologous primary vaccination scheme (1× Sinopharm/1× Pfizer) demonstrated a noteworthy protective effect. It is important to interpret this result with caution, as the number of individuals who received this scheme was relatively small (n = 197) compared with the other groups, although the difference reached statistical significance.

These findings align with several real-world effectiveness studies conducted globally that have consistently documented substantially lower protection by the different vaccines against infection with the Delta and Omicron variants compared with previous variants such as Alpha or the wild-type SARS-CoV-2 strain [28, 29, 33, 34]. COVID-19 vaccine efficacy was outpaced by viral mutations, explaining why various vaccines were highly effective against earlier variants but less so against Delta and Omicron variants. The emergence of these variants led to multiple waves, both locally and globally [35].

For instance, a nationwide Hungarian study evaluating VE during the Alpha wave found adjusted effectiveness against infection as follows: Pfizer: 83.3%; Sputnik V: 85.7%; AstraZeneca: 71.5%; and Sinopharm: 68.7% [33]. Similarly, a study from the UK observed high VE during the Alpha wave for Pfizer’s and AstraZeneca’s primary vaccination schemes but noted declines during the Delta wave [34].

In a nationwide Danish cohort study, mRNA vaccines were found to be highly effective against Delta variant infection but less so against Omicron, with VE estimates of 92% and 40%, respectively, during the first month after vaccination, in individuals aged < 60 years [29]. Similar results were reported in Belgium, where Pfizer’s VE was 96% against Alpha, 87% against Delta, and 31% against Omicron [28].

A recent systematic review and meta-analysis of studies on inactivated SARS-CoV-2 vaccines’ real-world effectiveness against infection revealed a pooled VE estimate of 53% against the Delta variant and a lower estimate of 16% against the Omicron variant, which circulated during 2021 and 2022 [36], consistent with our findings.

Early concerns about SARS-CoV-2 evolution focused on the potential emergence of variants with significant antigenic differences that could evade immunity acquired through vaccination or previous infection [37, 38]. Most widely utilized COVID-19 vaccines, which employ various platforms, as listed above, were initially developed based on the spike protein from early virus variants or the original wild-type virus [39].

While the Alpha variant exhibited limited antigenic changes, the Delta variant had a moderate ability to evade vaccine-induced antibodies [37, 38, 40, 41]. However, VE estimates remained acceptable against Delta [37, 38, 40, 41]. The Omicron variant, with its numerous antigenic sub-lineages, possesses a higher degree of mutation and significant antigenic drift, rendering it less neutralized by first-generation vaccines [37, 38, 40, 41]. Real-world data on VE against infections reflect these antigenic changes [37, 38, 40, 41].

In our cohort, booster doses were administered mostly 6 months after the second dose, coinciding with the transition from “Mixed” variants to the predominance of Omicron. VE generally increased, providing higher protection within three months after receiving a Pfizer booster. This trend was observed across primary vaccination schemes, but boosting with Sinopharm did not protect against infection in those initially vaccinated with Pfizer’s and Sinopharm’s primary schemes during Omicron phases. These results suggest that boosting is more effective with mRNA vaccines.

Consistent with our findings, a national study conducted in Malaysia compared the effectiveness of homologous and heterologous boosters. The results revealed that homologous inactivated vaccine boosting (VE = 33%) and adenovirus vaccine boosting (VE = 30%) were less effective than heterologous boosting (VE = 48%) and homologous boosting (VE = 51%) using Pfizer during the predominant Omicron period [42]. In addition, a previous study in Lebanon during the first Omicron wave showed similar effectiveness of mRNA boosters against infection, with VE measured at 64% and 57% for individuals who had previously received Pfizer’s and Sputnik V’s primary vaccination schemes, respectively [19].

### 4.3 Effect of time on VE against documented infection across the different vaccination schemes and variants

As previously discussed, vaccine-mediated protection against COVID-19 infection diminishes over time, particularly with the emergence of diverse SARS-CoV-2 variants such as Delta and Omicron. Consequently, booster doses have been introduced to strengthen and prolong immunity, with updated versions of COVID-19 vaccines targeting Omicron subvariants. In our cohort, changes in VE were evaluated from the Delta wave onwards. During the Delta wave and the phase characterized by “Mixed” variants, we observed fluctuations in VE estimates for Sinopharm’s and Pfizer’s primary vaccination schemes. VE declined between 3 to less than 6 months after the second dose but remained above 50%. However, VE of all primary schemes declined more rapidly during the Omicron phases, dropping below 20% at 3 to less than 6 months after the second dose and reaching null values at 6 months and beyond. Assessment of booster doses’ protection against infection was only possible during the second Omicron wave (BA.4/BA.5), which began in June 2022. The decline of booster VE with time was similarly steep for all homologous and heterologous booster schemes, with a slightly steeper decline observed for the homologous boosters from 3 to more than 6 months after the booster and beyond.

These findings align with real-world effectiveness reports consistently showing a significant decline in immunity against infection over time, with the most notable decrease observed with the Omicron variant compared to earlier variants such as Alpha or Delta [28, 29, 36]. In the previously mentioned pooled analysis of VE estimates of the primary schemes of inactivated vaccines, protection against SARS-CoV-2 infection decreased significantly after 6 months following primary vaccination, reaching 21% during the Delta wave and 7% during the Omicron wave [36]. For primary schemes of mRNA vaccines, the aforementioned Danish study reported waning immunity of the primary schemes across several variants, with effectiveness at 73.2% for Alpha, 50% for Delta, and 4% for Omicron, at more than 4 months since vaccination [29].

Regarding booster shots with inactivated vaccines for individuals who had received inactivated vaccines as their primary series, Kyaw and colleagues reported a complete loss of protection against infection just 2 months after the booster dose in a pooled analysis of 6 real-world effectiveness studies against the Omicron variant [43]. However, they also noted a less steep decline in VE against Omicron for BNT162b2 heterologous boosters in individuals who had initially received inactivated vaccines as their primary series. VE decreased from 57% in less than 3 months after the booster receipt to 35% thereafter [43].

Notably, the effectiveness estimate for the Sputnik V primary scheme increased from negative values within less than 3 months from the second dose to 57% within the 3 to less than 6 months following the second dose, particularly during the Omicron BA.1/BA.2 wave. Similarly, during the Omicron BA.4/BA.5 wave, VE estimates for the 2× Sputnik V/1× Pfizer regimen intriguingly rebounded from negative values within 3–<6 months after the booster to a remarkable 91% at 6 months and beyond. Dolzhikova and colleagues analyzed neutralizing antibody responses against variants of concern in sera samples of individuals vaccinated with Sputnik V, including those who were revaccinated with Sputnik Light [44]. Their study revealed that antibodies triggered by the Sputnik V vaccination matured and developed a broader capacity to neutralize emerging variants of concern within 6–9 months after the initial vaccination and 2–3 months after receiving a booster [44]. Similarly, a longitudinal study from Argentina demonstrated robust SARS-CoV-2 neutralizing antibodies and reduced viral variant escape to neutralization over time, particularly 6 months after primary vaccination with Sputnik V [45]. Investigators reported that antibodies produced in individuals vaccinated with Sputnik V exhibited increased cross-neutralization capacity against variants of concern as time passed [45]. Martynova and colleagues demonstrated that Sputnik V vaccination elicits a robust antibody response, recognizing diverse epitopes on the S-protein and a strong cellular response. These responses remain detectable for more than 3 months postvaccination, indicating the vaccine’s long-term efficacy [46].

### 4.4 VE against acute-care hospitalization

The significance of VE against hospitalization becomes apparent when VE estimates against infection for specific variants with particular vaccine protocols decline. Overall, our results revealed that VE estimates against hospitalization for primary vaccination schemes were generally lower during the Delta wave compared with the Alpha wave. However, all primary schemes remained highly protective, with effectiveness above 80% against hospitalization across all postvaccination timeframes. During the phase characterized by the presence of “Mixed” Delta and Omicron variants and continuing thereafter during the Omicron-predominant waves, sharp declines in effectiveness against acute-care hospitalization were observed for primary vaccination schemes. Booster schemes provided full protection against hospitalization during the “Mixed” phase of the Delta and Omicron variants, but a decline was noted in booster protection when the Omicron BA.1/BA.2 variant predominated. Notably, all primary and booster vaccination schemes provided 100% protection against critical illness and mortality during all waves in our cohort. Contrary to the common belief that effectiveness against infection wanes over time while effectiveness against hospitalization lasts, our data has shown that effectiveness against hospitalization also declines with time across all primary and booster schemes, especially during Omicron predominance.

The time-sensitive waning of VE against hospitalization was observed against Delta infection-induced hospitalization, but it is dramatically accelerated against Omicron infection-induced hospitalization [47]. In an observational prospective case-control study involving 21 hospitals in the US, Pfizer’s primary series was effective against both Alpha and Delta variant-associated hospitalization, with VE measuring 85% [48]. However, lower protection was observed against Omicron-associated hospitalization, reaching 65% [48]. mRNA boosters increased effectiveness against Delta and Omicron-induced hospitalizations to 94% and 86%, respectively [48]. In a Brazilian nationwide case-control study, the effectiveness of the CoronaVac primary series against COVID-19 associated hospitalization was substantially lower during an Omicron-dominated period (56%) compared with a Delta-dominated period (87%) [49]. During the Omicron-dominated period, a homologous CoronaVac booster dose conferred a smaller increase in protection against severe disease (74%) compared with a heterologous Pfizer booster (86%) [49]. Notably, the increased protection afforded by a homologous booster against severe disease waned during the 4 months after its administration, in contrast to the durable effectiveness of the heterologous Pfizer booster [49].

VE against infection exhibited statistically significant negative values within both primary and booster vaccination schemes during the periods when the Omicron variant predominated. However, it is crucial to note that these negative estimated values of effectiveness likely reflect the influence of certain factors rather than indicating a true negative biological effectiveness of the vaccines [50, 51]. These factors include vaccinated individuals having a higher risk of network-level exposure, increased social contact rates, greater susceptibility, lower rates of prior infection, or less adherence to safety measures and barriers such as mask usage, hand hygiene, and social distancing compared with unvaccinated individuals [50, 51]. In addition, over time, the number of vaccinated individuals increased, along with those previously exposed to the virus, leaving unvaccinated, COVID-naïve adults as a minority, especially during later waves of the pandemic [52–54]. These factors significantly impact vaccine’s effectiveness against recent variants like Omicron compared with earlier variants.

Based on our data, which demonstrates that vaccine protection against critical illness and death in a generally young and healthy population remains preserved over time, frequent booster shots may not be necessary for this group, particularly those who can tolerate infection without severe complications. This is especially pertinent as VE against infection tends to diminish with time and with the emergence of new variants. We propose that young and healthy individuals, such as those included in this cohort, may not require regular vaccine boosters once they have established protection against critical illness and death through primary vaccination. Their immunity can potentially be naturally reinforced, akin to the immune response historically seen with common colds before the COVID-19 pandemic. However, this conclusion may not apply to more vulnerable individuals, such as those with comorbidities or immune deficiencies that impede the initial vaccine response. Separate studies should focus on these individuals to assess VE against infection, hospitalization, critical illness, or COVID-19-associated mortality. This will enable evidence-based decisions regarding the optimal vaccine regimens and booster schedules.

### 4.5 Immunogenicity of vaccination schemes involving Sinopharm and Pfizer

In primary vaccination schemes, the homologous Pfizer regimen elicited a higher humoral response compared to Sinopharm. However, the humoral response generated by the homologous Pfizer regimen was comparable to that of the heterologous primary regimen 1× Sinopharm/1× Pfizer. Over time, we observed a decline in antispike IgG GMT for both Sinopharm’s and Pfizer’s primary vaccination schemes. Pfizer’s booster dose elicited a similarly robust humoral response in individuals who initially received either Pfizer’s or Sinopharm’s primary vaccination schemes. However, a Sinopharm booster dose induced a significantly lower humoral response compared to the latter booster schemes in individuals who had initially received either Pfizer or Sinopharm as their primary vaccination.

These findings are consistent with results from other immunogenicity studies in the literature, where antispike IgG GMT levels were consistently higher among recipients of the primary Pfizer vaccine scheme compared to those who received Sinopharm [55, 56]. Humoral immunity was observed to be short-lived and tended to decline over time, as documented in other studies [57–59]. Heterologous boosting has been explored in individuals who previously received primary inactivated vaccine schemes. In a prospective cohort study evaluating the immunogenicity of Pfizer’s booster among Peruvian healthcare workers who had previously received the primary Pfizer or Sinopharm vaccination schemes, investigators reported that the antispike IgG GMT levels produced by the heterologous vaccine regimen was significantly higher than that elicited by the homologous booster regimen [56].

The concept of an immune correlate of protection (or immuno-bridging) aims to utilize immunological measurements as predictive markers for protection against infectious diseases [37]. Before the emergence of variants that could evade vaccine efficacy, serology testing and the stratification of seroconversion levels proved useful in promptly identifying high-risk groups of vaccine non-responders. These groups may not develop a viral neutralizing response, even if they show seroconversion, and therefore could remain at a higher risk of infection despite vaccination [60].

Throughout the pandemic, studies have consistently indicated a strong relationship between high titers of antispike RBD IgG and a less severe disease state, as well as a lower incidence of breakthrough infections. Higher IgG binding has been associated with increased protection against infection with the wild-type, Alpha, and Delta variant strains of the SARS-CoV-2 virus [61–64]. Furthermore, a robust correlation has been observed between antispike RBD IgG titers and neutralizing antibody titers, suggesting that IgG concentrations provide insights into protection against infection with the ancestral strain or relatively homologous variants of concern [64–66].

In our study, Pfizer vaccine elicited a significantly higher immune response compared to Sinopharm after initial vaccination. Nevertheless, both vaccines demonstrated high levels of protection against infection caused by the Alpha and Delta variants, but their effectiveness gradually decreased and diverged from each other when it came to the Omicron variants.

Consistent with our findings, a study from Thailand by Takheaw and colleagues determined the correlations between the levels of anti-RBD IgG and neutralizing antibodies against SARS-CoV-2 variants in vaccinated individuals with two doses of CoronaVac or one dose each of CoronaVac and AstraZeneca [67]. The investigators reported a high correlation between anti-RBD IgG and neutralizing antibodies against the wild-type, Alpha, and Delta variants but not with the Omicron variant [67]. Among individuals with high levels of anti-RBD IgG, 93% had neutralizing antibodies against the wild-type, Alpha, and Delta variants, but none had neutralizing antibodies against Omicron [67]. The study concluded that anti-RBD IgG levels cannot predict the presence of neutralizing antibodies against the Omicron variant [67].

In a study from Sweden, Vikström and colleagues found no indication that levels of vaccine-induced antibodies after an mRNA booster dose protected against infection with the Omicron variant [68]. They found no pattern supporting that higher levels of circulating S-binding IgG protected against SARS-CoV-2 infection [68]. They attributed their findings to the Omicron variant’s significant escape from neutralizing antibodies [68].

### 4.6 Limitations

This study, relying on real-time data, encountered several limitations. First, there were instances of missing data, particularly those regarding the progression of VE against specific variants over time within defined time points and for particular vaccination schemes. This limitation arose from the retrospective collection of data during different waves of the pandemic, with the follow-up period coinciding with subsequent surge in cases related to other emerging variants.

Second, a relatively young and healthy cohort was studied, which may restrict the generalizability of our findings to the entire Lebanese population. However, this demographic characteristic can also be viewed as a strength, as it allows for the study of effectiveness while controlling for confounding factors such as age and comorbidities.

The detection of laboratory-confirmed infections relied on SARS-CoV-2 PCR testing of symptomatic and asymptomatic individuals during contact tracing. Ideally, systematic screening at multiple time intervals would have allowed us to detect more asymptomatic infections. Nevertheless, our study was based on real-world data in a low-income country with limited resources, facing a socioeconomic crisis. The bias toward including only symptomatic infections rather than all infections was minimized by the vigilant contact tracing system established by the MoD since the start of the pandemic. In addition, the definition of variant-specific cases was based on epidemiologic evidence rather than genomic testing of the variants in the studied population.

The negative estimated effectiveness values during the Omicron waves were more likely a result of bias rather than a genuine indication of the vaccines having negative biological effectiveness. This bias may have arose from factors such as the gradual reduction of the unvaccinated population without prior COVID-19 exposure over time and the increased exposure of vaccinated individuals to the virus. In addition, there may be unequal compliance with safety measures when compared with the unvaccinated population. These factors can lead to an underestimation of VE [69].

It is important to note that the sample size for VE against COVID-19 acute-care hospitalization during Omicron periods was limited in this study. While every effort was made to include all eligible cases, the small sample size during these periods may affect the generalizability of our findings. Thus, caution should be exercised when interpreting the results from these time periods.

The lack of cellular immunity and serum-neutralizing antibody testing in the immunogenicity evaluation was a limitation. Notably, humoral immunity was not assessed for vaccination schemes including Sputnik V or AstraZeneca vaccines. Furthermore, assessing changes in humoral response beyond 3 months post-first booster in any scheme was not possible.

Despite these limitations, our study offers a comprehensive view of real-world effectiveness for different homologous and heterologous primary and booster vaccination schemes against various vaccine-tolerant variants, specifically during periods of high viral circulation rather than throughout the entire wave.

## 5. Conclusion

In this case-control study, we observed consistent high effectiveness of COVID-19 vaccines during the pre-Omicron waves, with the duration since vaccination and the emergence of new variants of concern playing pivotal roles in real-world effectiveness. VE estimates for primary vaccination schemes during the Delta wave were generally lower than those reported during the Alpha wave, yet they provided significant protection against infection. However, VE estimates showed a consistent decline for the Omicron variant across all post-vaccination timeframes and for all combinations of primary and initial booster schemes examined. Despite this decline, none of the breakthrough infections in any vaccinated group progressed to critical or fatal COVID-19 during the study period.

In terms of immunogenicity analysis, the homologous Pfizer regimen elicited a stronger humoral response than Sinopharm, while the heterologous 1× Sinopharm/1× Pfizer primary regimen produced comparable results to the homologous Pfizer regimen. Both Sinopharm and Pfizer primary vaccination schemes showed a decrease in humoral immunity titers over time. Boosting with Pfizer after a primary Pfizer or Sinopharm vaccination induced a significant increase in humoral immunity, whereas Sinopharm acted as a weaker booster for immunity.

While our results offer valuable insights into VE in healthy young adults, it is crucial to recognize that conclusions about vaccination and booster strategies may differ in older and immunocompromised individuals. Future studies involving these groups will provide additional insights. Continuous research and monitoring are essential to comprehensively understand vaccine-mediated immune responses, particularly during pandemics, and will be crucial for our preparedness in facing future health crises.

## Supporting information

S1 Table STROBE statement. (DOCX)

S2 Table Demographic and clinical characteristics of the participants used to estimate effectiveness of the different vaccination schemes during the Alpha variant wave. Abbreviations: PFZ = Pfizer-BioNTech (BNT162b2), SPh = Sinopharm (BBIBP-CorV).
(XLSX)

S3 Table Demographic and clinical characteristics of the participants used to estimate effectiveness of the different vaccination schemes during the Delta variant wave. Abbreviations: AZ = AstraZeneca (ChAdOx1 nCoV-19), PFZ = Pfizer-BioNTech (BNT162b2), SPh = Sinopharm (BBIBP-CorV), SPTV = Gamaleya Sputnik V (Gam-COVID-Vac).
(XLSX)

S4 Table Demographic and clinical characteristics of the participants used to estimate effectiveness of the different vaccination schemes during the mixed Delta and Omicron variant wave. Abbreviations: AZ = AstraZeneca (ChAdOx1 nCoV-19), PFZ = Pfizer-BioNTech (BNT162b2), SPh = Sinopharm (BBIBP-CorV), SPTV = Gamaleya Sputnik V (Gam-COVID-Vac).
(XLSX)

S5 Table Demographic and clinical characteristics of the participants used to estimate effectiveness of the different vaccination schemes during the Omicron BA.1/BA.2 variant wave. Abbreviations: AZ = AstraZeneca (ChAdOx1 nCoV-19), PFZ = Pfizer-BioNTech (BNT162b2), SPh = Sinopharm (BBIBP-CorV), SPTV = Gamaleya Sputnik V (Gam-COVID-Vac).
(XLSX)

S6 Table Demographic and clinical characteristics of the participants used to estimate effectiveness of the different vaccination schemes during the Omicron BA.4/BA.5 variant wave. Abbreviations: AZ = AstraZeneca (ChAdOx1 nCoV-19), PFZ = Pfizer-BioNTech (BNT162b2), SPh = Sinopharm (BBIBP-CorV), SPTV = Gamaleya Sputnik V (Gam-COVID-Vac).
(XLSX)

S7 Table Demographic data of participants in immunogenicity subgroup analyses. Abbreviations: mo = months, PFZ = Pfizer-BioNTech (BNT162b2), SPh = Sinopharm (BBIBP-CorV).
(DOCX)
