# Supplementary material for: Immunogenicity and real-world effectiveness of COVID-19 vaccines in Lebanon: Insights from primary and booster schemes, variants, infections, and hospitalization
Source: PLoS One. 2024 Sep 13;19(9):e0306457. doi: 10.1371/journal.pone.0306457 (PMC11398646; doi:10.1371/journal.pone.0306457)
Supplement: S1 Table — (DOCX) [file pone.0306457.s001.docx]

**S1 Table.** STROBE Statement

|  | Item No. | Recommendation | Page  No. | Relevant text from manuscript |
| --- | --- | --- | --- | --- |
| **Title and abstract** | 1 | (*a*) Indicate the study’s design with a commonly used term in the title or the abstract | Page 1, page 3, | Lines 49-50 |
|  |  | (*b*) Provide in the abstract an informative and balanced summary of what was done and what was found | Pages 3-4 | Lines 49-76 |
| Introduction | | | |  |
| Background/rationale | 2 | Explain the scientific background and rationale for the investigation being reported | Pages 5-6 | Lines 99-145 |
| Objectives | 3 | State specific objectives, including any prespecified hypotheses | Pages 6-7 | Lines 147-156 |
| Methods | | | |  |
| Study design | 4 | Present key elements of study design early in the paper | Page 8 | Lines 174-184 |
| Setting | 5 | Describe the setting, locations, and relevant dates, including periods of recruitment, exposure, follow-up, and data collection | Page 9-14 | Lines 187-332 |
| Participants | 6 | (*a*) *Cohort study*—Give the eligibility criteria, and the sources and methods of selection of participants. Describe methods of follow-up  *Case-control study*—Give the eligibility criteria, and the sources and methods of case ascertainment and control selection. Give the rationale for the choice of cases and controls  *Cross-sectional study*—Give the eligibility criteria, and the sources and methods of selection of participants | Pages 9, 11, 12, 13, | Lines 187-233, 248-257, 291-299 |
|  |  | (*b*) *Cohort study*—For matched studies, give matching criteria and number of exposed and unexposed  *Case-control study*—For matched studies, give matching criteria and the number of controls per case | Not applicable | Not applicable |
| Variables | 7 | Clearly define all outcomes, exposures, predictors, potential confounders, and effect modifiers. Give diagnostic criteria, if applicable | Pages 8, 11, 12, 13 | Lines 187-196, 248-257, 291-303, 302-313 |
| Data sources/ measurement | 8* | For each variable of interest, give sources of data and details of methods of assessment (measurement). Describe comparability of assessment methods if there is more than one group | Page 9,10,11, 13,14 | Lines 198-205, 226-233, 248-257, 291-303, 319-333 |
| Bias | 9 | Describe any efforts to address potential sources of bias | Pages 9-12 | Lines 198-200, 235-299, the whole study population involving cases and controls were mostly young male healthy adults for vaccine effectiveness analysis and for immunogenicity analysis |
| Study size | 10 | Explain how the study size was arrived at | Not applicable | The whole cohort data was used |

Continued on next page

| Quantitative variables | 11 | Explain how quantitative variables were handled in the analyses. If applicable, describe which groupings were chosen and why | Pages 16-17 | 392-399 |
| --- | --- | --- | --- | --- |
| Statistical methods | 12 | (*a*) Describe all statistical methods, including those used to control for confounding | Page 14-16 | Lines 352-404 |
|  |  | (*b*) Describe any methods used to examine subgroups and interactions | Not applicable | Not applicable |
|  |  | (*c*) Explain how missing data were addressed | Vaccination group specified and data not included | Vaccination group specified and data not included |
|  |  | (*d*) *Cohort study*—If applicable, explain how loss to follow-up was addressed  *Case-control study*—If applicable, explain how matching of cases and controls was addressed  *Cross-sectional study*—If applicable, describe analytical methods taking account of sampling strategy | Not applicable | Not applicable |
|  |  | (*e*) Describe any sensitivity analyses | Not applicable | Not applicable |
| Results | | | | |
| Participants | 13* | (a) Report numbers of individuals at each stage of study—eg numbers potentially eligible, examined for eligibility, confirmed eligible, included in the study, completing follow-up, and analysed | Pages 17-19, 39 | Lines 407-448, plus figure-2, table 4 |
|  |  | (b) Give reasons for non-participation at each stage | Page 17 | figure-2 |
|  |  | (c) Consider use of a flow diagram | Page 17 | figure-2 |
| Descriptive data | 14* | (a) Give characteristics of study participants (eg demographic, clinical, social) and information on exposures and potential confounders | Pages 17, 38 | Lines 422-426, 815-818, S2-S7 tables |
|  |  | (b) Indicate number of participants with missing data for each variable of interest | Vaccination group specified and data not included | Vaccination group specified and data not included |
|  |  | (c) *Cohort study*—Summarise follow-up time (eg, average and total amount) | Not applicable | Not applicable |
| Outcome data | 15* | *Cohort study*—Report numbers of outcome events or summary measures over time | Page 39 | Table 4 |
|  |  | *Case-control study—*Report numbers in each exposure category, or summary measures of exposure | Pages 17, 20-22, 30-32 | Figure 2, table 2, table 3 |
|  |  | *Cross-sectional study—*Report numbers of outcome events or summary measures | Not applicable | Not applicable |
| Main results | 16 | (*a*) Give unadjusted estimates and, if applicable, confounder-adjusted estimates and their precision (eg, 95% confidence interval). Make clear which confounders were adjusted for and why they were included | Page 15, 16  Page 18-38 | Lines 376-390  Lines 449-811, table 2, table 3 |
|  |  | (*b*) Report category boundaries when continuous variables were categorized | Page 39 | Table 4 |
|  |  | (*c*) If relevant, consider translating estimates of relative risk into absolute risk for a meaningful time period | Not applicable | Not applicable |

Continued on next page

| Other analyses | 17 | Report other analyses done—eg analyses of subgroups and interactions, and sensitivity analyses | Not applicable | Not applicable |
| --- | --- | --- | --- | --- |
| Discussion | | | | |
| Key results | 18 | Summarise key results with reference to study objectives | Page 45-55 | Lines 1020-1032, 1084-1096, 1115-1127, 1150-1156, 1175-1191, 1252-1261 |
| Limitations | 19 | Discuss limitations of the study, taking into account sources of potential bias or imprecision. Discuss both direction and magnitude of any potential bias | Page 57-59 | Lines 1310-1352 |
| Interpretation | 20 | Give a cautious overall interpretation of results considering objectives, limitations, multiplicity of analyses, results from similar studies, and other relevant evidence | Page 53-55 | Lines 1215-1248 |
| Generalisability | 21 | Discuss the generalisability (external validity) of the study results | Pages 45, 58, 60 | Lines 1003-1016, 1318-1322, 1374-1381 |
| Other information | |  | | |
| Funding | 22 | Give the source of funding and the role of the funders for the present study and, if applicable, for the original study on which the present article is based | Page 58 | Lines 1306-1309 |

*Give information separately for cases and controls in case-control studies and, if applicable, for exposed and unexposed groups in cohort and cross-sectional studies.
