# Supplementary material for: Immunogenicity and real-world effectiveness of COVID-19 vaccines in Lebanon: Insights from primary and booster schemes, variants, infections, and hospitalization
Source: PLoS One. 2024 Sep 13;19(9):e0306457. doi: 10.1371/journal.pone.0306457 (PMC11398646; doi:10.1371/journal.pone.0306457)
Supplement: S7 Table — Abbreviations: mo = months, PFZ = Pfizer-BioNTech (BNT162b2), SPh = Sinopharm (BBIBP-CorV). (DOCX) [file pone.0306457.s007.docx]

**Table S7-**Demographic data of participants in immunogenicity subgroup analyses

|  | 2 × SPh (< 3 mo) | 2 × SPh (≥ 6 mo) | 1 × SPh/1 × PFZ (< 3 mo) | 2 × SPh/1 × SPh (< 3 mo) | 2 × SPh/1 × PFZ (< 3 mo) | 2 × PFZ (< 3 mo) | 2 × PFZ (3 to < 6 mo) | 2 × PFZ (≥ 6 mo) | 2 × PFZ/1 × PFZ (< 3 mo) | 2 × PFZ/1 × SPh (< 3 mo) |
| --- | --- | --- | --- | --- | --- | --- | --- | --- | --- | --- |
| Number of Participants | 20 | 228 | 56 | 222 | 228 | 48 | 26 | 55 | 55 | 43 |
| Age (years) |  |  |  |  |  |  |  |  |  |  |
| <50 | 20 (100%) | 228 (100%) | 56 (100%) | 222 (100%) | 228 (100%) | 45 (93.75%) | 21 (80.8%) | 52 (94.5%) | 52 (94.5%) | 42 (97.7%) |
| ≥ 50 to < 65 | 0 | 0 | 0 | 0 | 0 | 3 (6.25%) | 5 (19.2%) | 3 (5.6%) | 3 (5.6%) | 1 (2.3%) |
| Gender |  |  |  |  |  |  |  |  |  |  |
| Male | 20 (100%) | 227 (99.6%) | 56 (100%) | 214 (99.4%) | 227 (99.6%) | 38 (79.2%) | 14 (53.8%) | 37 (67.3%) | 37 (67.3%) | 32 (74.4%) |
| Female | 0 | 1 (0.4%) | 0 | 8 (0.6%) | 1 (0.4%) | 10 (20.8%) | 12 (46.2%) | 18 (32.7%) | 18 (32.7%) | 11 (25.6%) |

**Abbreviations:** mo = months, PFZ = Pfizer-BioNTech (BNT162b2), SPh = Sinopharm (BBIBP-CorV).
